# Genomes reveal marked differences in the adaptive evolution between orangutan species

**DOI:** 10.1186/s13059-018-1562-6

**Published:** 2018-11-15

**Authors:** Maja P. Mattle-Greminger, Tugce Bilgin Sonay, Alexander Nater, Marc Pybus, Tariq Desai, Guillem de Valles, Ferran Casals, Aylwyn Scally, Jaume Bertranpetit, Tomas Marques-Bonet, Carel P. van Schaik, Maria Anisimova, Michael Krützen

**Affiliations:** 1 Evolutionary Genetics Group, Department of Anthropology, University of Zurich, Winterthurerstrasse 190, 8057 Zürich, Switzerland; 2 Department of Evolutionary Biology and Environmental Studies, University of Zurich, Winterthurerstrasse 190, 8057 Zürich, Switzerland; 3 Swiss Institute of Bioinformatics, Quartier Sorge - Batiment Genopode, 1015 Lausanne, Switzerland; 4 Lehrstuhl für Zoologie und Evolutionsbiologie, Department of Biology, University of Konstanz, Universitätsstrasse 10, 78457 Konstanz, Germany; 5 Institut de Biologia Evolutiva (UPF-CSIC), Universitat Pompeu Fabra, Doctor Aiguader 88, 08003 Barcelona, Spain; 6 Department of Genetics, University of Cambridge, Downing Street, Cambridge, CB2 3EH UK; 7 Servei de Genòmica, Universitat Pompeu Fabra, Doctor Aiguader 88, 08003 Barcelona, Spain; 8 Institute of Evolutionary Biology (UPF-CSIC), Universitat Pompeu Fabra, Doctor Aiguader 88, Barcelona, Spain; 9 Catalan Institution of Research and Advanced Studies (ICREA), Passeig de Lluís Companys 23, Barcelona, Spain; 10 CNAG-CRG, Centre for Genomic Regulation (CRG), Barcelona Institute of Science and Technology (BIST), Baldiri i Reixac 4, Barcelona, Spain; 11 Institut Català de Paleontologia Miquel Crusafont, Universitat Autònoma de Barcelona, Edifici ICTA-ICP, c/ Columnes s/n, Cerdanyola del Vallès, Barcelona, Spain; 12 Institute of Applied Simulations, School of Life Sciences and Facility Management, Zurich University of Applied Sciences ZHAW, Einsiedlerstrasse 31a, 8820 Wädenswil, Switzerland

**Keywords:** Local adaptation, Great apes, Demographic history, Cognitive evolution, *Pongo*, Pleistocene glaciations

## Abstract

**Background:**

Integrating demography and adaptive evolution is pivotal to understanding the evolutionary history and conservation of great apes. However, little is known about the adaptive evolution of our closest relatives, in particular if and to what extent adaptions to environmental differences have occurred. Here, we used whole-genome sequencing data from critically endangered orangutans from North Sumatra (*Pongo abelii*) and Borneo (*P*. *pygmaeus*) to investigate adaptive responses of each species to environmental differences during the Pleistocene.

**Results:**

Taking into account the markedly disparate demographic histories of each species after their split ~ 1 Ma ago, we show that persistent environmental differences on each island had a strong impact on the adaptive evolution of the genus *Pongo*. Across a range of tests for positive selection, we find a consistent pattern of between-island and species differences. In the more productive Sumatran environment, the most notable signals of positive selection involve genes linked to brain and neuronal development, learning, and glucose metabolism. On Borneo, however, positive selection comprised genes involved in lipid metabolism, as well as cardiac and muscle activities.

**Conclusions:**

We find strikingly different sets of genes appearing to have evolved under strong positive selection in each species. In Sumatran orangutans, selection patterns were congruent with well-documented cognitive and behavioral differences between the species, such as a larger and more complex cultural repertoire and higher degrees of sociality. However, in Bornean orangutans, selective responses to fluctuating environmental conditions appear to have produced physiological adaptations to generally lower and temporally more unpredictable food supplies.

**Electronic supplementary material:**

The online version of this article (10.1186/s13059-018-1562-6) contains supplementary material, which is available to authorized users.

## Background

Local adaptations are expected when species colonize novel environments, or within the context of heterogeneous environments and population structure [1]. In humans, paleoclimatic changes may have triggered the expansion out of Africa into novel environments (e.g., [2, 3]). These migrations through the Late Pleistocene and the Neolithic exposed our ancestors to novel selective pressures, leading to numerous local adaptations (reviewed in [4]). Examples for such adaptations are thermoregulation [5] and lighter skin pigmentation in response to living at higher latitudes [6, 7], tolerance to hypoxia in higher altitudes [8–10], and complex traits like body size [11]. In the Neolithic, with the switch to agricultural subsistence, cattle-breeding, higher population densities, and increased infectious-disease loads lead to changes in phenotypes linked to immune responses [12–14]. Compared to humans, however, little is known about the adaptive evolution in non-human great apes [15–18] and whether or to what extent adaptations to environmental changes have occurred. Identifying genetic signatures of adaptive evolution in our closest relatives will not only enhance our understanding of how adaptation has shaped genetic variation in extant great ape populations, but may also help us to understand the differences between human and non-human great apes.

**Fig. 1:**
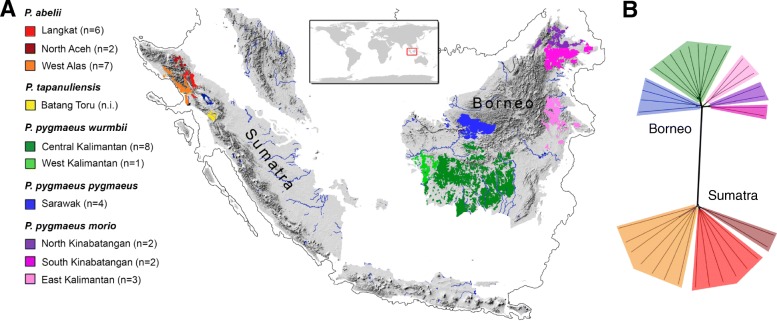
Distribution and population structure of the genus *Pongo*. **a** Sampling areas across the current distribution of orangutans (adapted from Nater et al. 2017 [33]). The thin gray line indicates the extent of the exposed Sunda shelf during the last glacial maximum (19–26 ka, ~ 120 m below current sea level). Numbers of sequenced individuals are provided in the legend. We did not include (n.i.) the recently described *P*. *tapanuliensis* [33], as the low sample size of available genomes (*n* = 2) currently restricts such analyses. **b** Neighbor-net phylogenetic network of the 35 orangutan samples. Color codes of the populations correspond to those of **a**

Orangutans (genus: *Pongo*) are the only great apes in Asia and phylogenetically the most distantly related to humans. Once widely distributed throughout Southeast Asia [19], their range is currently restricted to increasingly isolated forest patches on northern Sumatra (*P*. *abelii* and *P*. *tapanuliensis*) and Borneo (*P*. *pygmaeus*) (Fig. 1) [20]. During most of the Pleistocene, orangutans on the two islands have been subjected to markedly different environmental conditions. On northern Sumatra, where all extant Sumatran orangutans occur, habitat conditions have been generally more stable and productive due to volcanism-enriched soils, lower levels of cloud cover, and more regular precipitation patterns [21, 22]. On Borneo, however, fluctuations of rainforest coverage during glacial cycles were more pronounced [23, 24]. Furthermore, particularly in the northeastern part of Borneo, orangutans (*P*. *p*. *morio*) have been exposed to marked temporal fluctuations in fruit availability, including mast fruiting events with short periods of overabundance of fruit, followed by extended periods of low fruit production [22, 25–27]. Moreover, severe and unpredictable droughts and forest fires due to El Niño events have been causing prolonged periods of extreme food scarcity, posing major threats to orangutan survival in this region [28, 29]. As a consequence of these differences in forest productivity and temporal stability of food supply, population densities are generally higher on northern Sumatra compared to Borneo and particularly low in the northeast of Borneo [30, 31].

In line with these long-term environmental differences, orangutans on Borneo and Sumatra show well-documented geographic variation in their biology, including morphology, physiology, life history, and cultural repertoire [32]. Orangutans therefore provide a unique opportunity to investigate the interplay between environmental processes, demography, and adaptive evolution in two closely related, yet phenotypically distinct great ape species.

To identify molecular targets of adaptive evolution, we used data from 35 complete orangutan genomes from nine geographically distinct sampling areas (Fig. 1) across the range of Bornean (*P*. *pygmaeus*) and North Sumatran (*P*. *abelii*) orangutans. We did not include the recently described Tapanuli orangutan (*P*. *tapanuliensis*) [33], as the low sample size (*n* = 2) of available genomes impeded such analyses.

We carried out multiple tests to detect different molecular patterns associated with positive selection, such as distortion of the site-frequency spectrum [34, 35], extended haplotype structure [36, 37], and substitution patterns in coding regions [38, 39]. Detecting signatures of past and ongoing selective processes on the background of complex demographic histories is challenging [40, 41], as population size changes and structure can produce patterns similar to the ones expected under non-neutral scenarios [34]. To avoid the detection of spurious signatures of selection, reliable identification of the targets of selection requires detailed knowledge about the demographic history [42]. We therefore used a detailed demographic model [33] to account for outliers in genome scans caused by neutral demographic processes.

## Results

### Demographic history of the genus *Pongo*

**Fig. 2:**
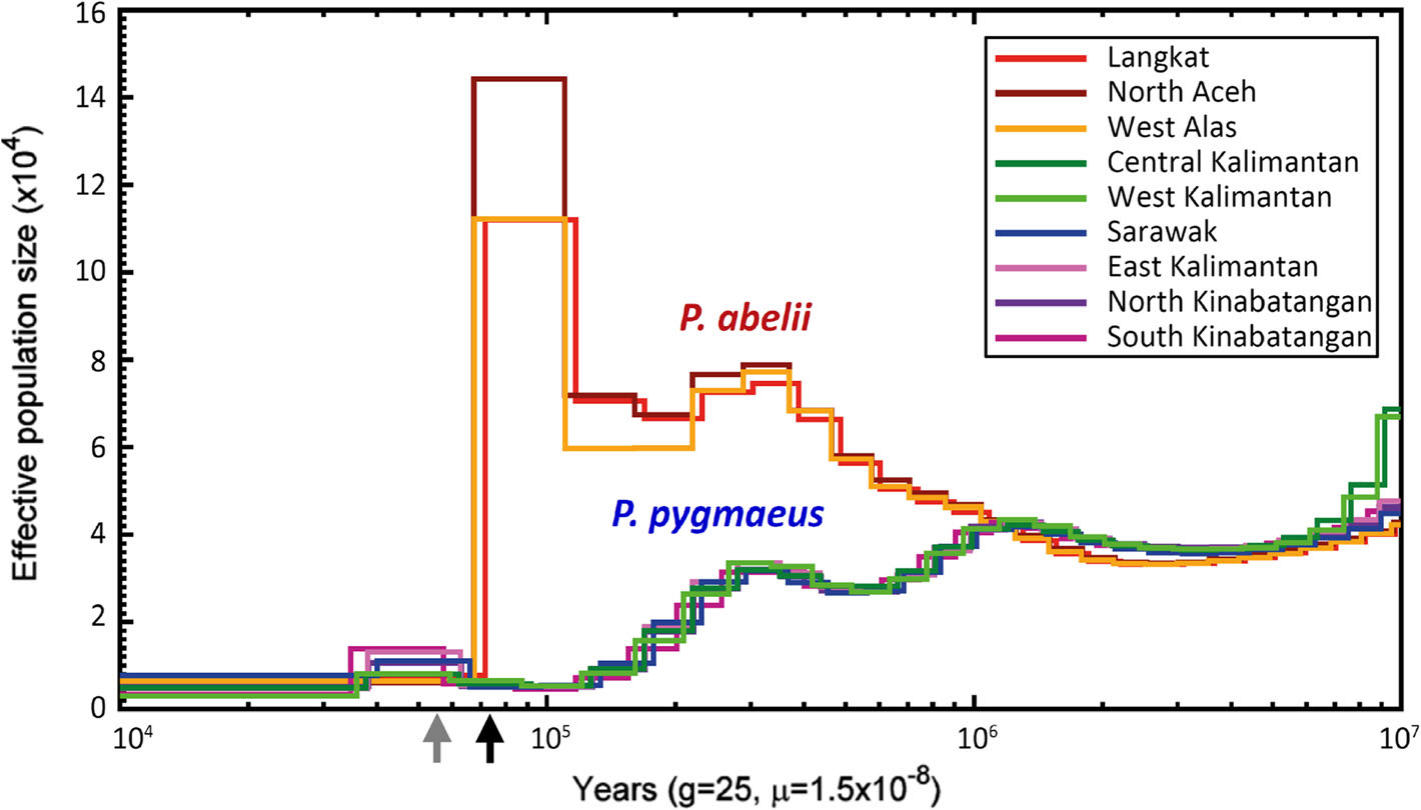
Autosomal *N*_e_ history inferred by pairwise sequentially Markovian coalescent (PSMC) analysis. For each orangutan sampling area, one high-coverage (≥ 20x) genome is plotted. Color codes match those of Fig. 1. The *x*-axis shows time scaled in years, assuming a generation time of 25 years and an autosomal mutation rate of 1.5 × 10^−8^ per site per generation. The gray arrow indicates the arrival of modern humans on Sundaland (~ 60–50 ka) [61], the black arrow shows the Toba supereruption (~ 73 ka) [59]

**Fig. 3:**
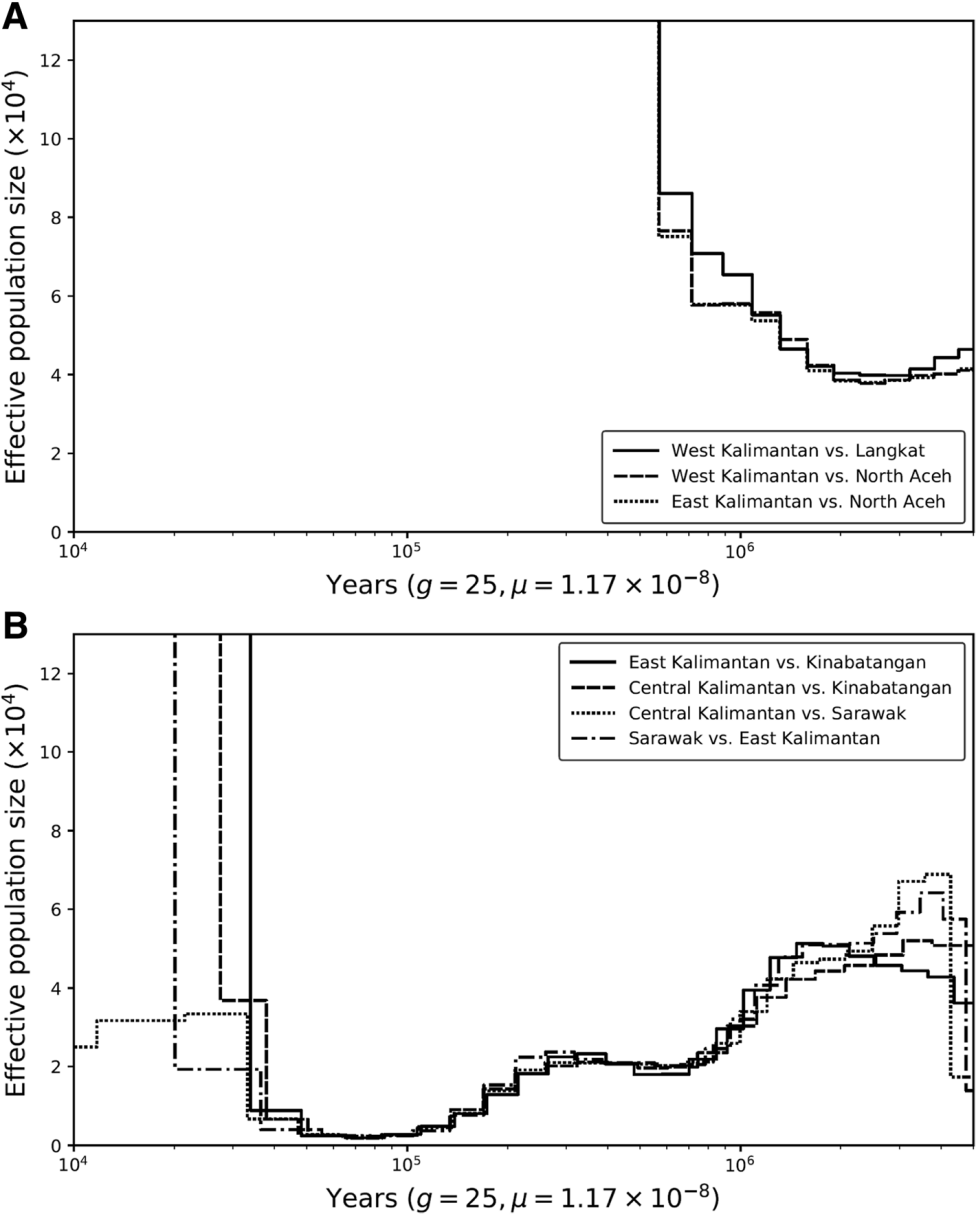
Gene flow across time in the genus *Pongo*. **a** Temporal estimates of cross-population *N*_e_ between population pairs from multiple sequentially Markovian coalescent (MSMC) analyses by comparing X-chromosomal haplotypes of Sumatran populations of Langkat and North Aceh to the Bornean populations of Central/West Kalimantan and East Kalimantan. Cross-population *N*_e_ is inversely proportional to gene flow between population pairs, but also influenced by within-population *N*_e_. The *x*-axis shows time scaled in years, assuming a generation time of 25 years and a X-chromosomal mutation rate of 1.17 × 10^−8^ per site per generation. **b** Cross-population *N*_e_ between Bornean populations estimated from X-haplotypes in MSMC, revealing complete genetic isolation from 30 to 10 ka onwards for all population pairs except Central/West Kalimantan and Sarawak

We investigated the demographic history of the Bornean and Sumatran orangutan populations using the pairwise sequentially Markovian coalescent (PSMC) [43] and multiple sequentially Markovian coalescent (MSMC2) [44] methods. While PSMC (no phasing required) infers variation in effective population size (*N*_e_) over time based on single diploid genomes, MSMC2 (phasing required) can additionally infer temporal patterns of gene flow by comparing haplotype pairs across populations. However, inaccurate phasing of autosomal data will break up long haplotype segments, biasing split time estimates and masking signatures of recent gene flow [45]. We aimed to avoid such issues by focusing on male haploid X chromosomes. Our analyses suggested that the ancestors of Bornean and Sumatran orangutans began to diverge ~ 1 Ma (Fig. 2), and that gene flow between the diverged populations ceased between 1 Ma and 400 ka (Fig. 3a), with no evidence of subsequent gene flow. However, as X chromosomes in great apes have been found to be affected by widespread selection [46, 47], potential biases in coalescent-based estimates of *N*_e_ could arise using this approach. We therefore repeated the gene flow analyses using entire phased autosomal genomes, resulting in highly congruent estimates of the divergence time between orangutan species (Additional file 1: Figure S7A).

To further corroborate the gene flow estimates and assess the impact of population structure on the PSMC and MSMC2 analyses, we fitted a demographic model to the empirical multi-dimensional site-frequency spectrum using the program momi2 [48] (Additional file 1: Figure S8). Most parameter estimates were consistent with the findings from PSMC and MSMC2 (Additional file 1: Table S4). We obtained a split time between Borneo and Sumatra of ~ 731 ka (95% CI 716–741 ka), which falls right into the time window when the cross-population *N*_e_ estimates from MSMC2 increased towards infinity (Additional file 1: Figure S7A). However, while there was no indication of gene flow between Sumatra and Borneo more recent than ~ 400 ka in the MSMC2 analysis, our momi2 analysis suggested a more recent phase of admixture during the last glacial period (~ 44 ka, 40–48 ka). This admixture pulse was asymmetrical, with ~ 4.2% (4.0–4.4%) of Sumatran lineages being derived from Borneo while only ~ 1.5% (1.3–1.7%) of the Bornean lineages exhibit recent Sumatran ancestry.

After their initial separation in the Middle Pleistocene, orangutan populations on Borneo and Sumatra exhibited strongly divergent demographic trajectories (Fig. 2). While Sumatran orangutans went through a continuous increase in *N*_e_ until ~ 70 ka ago, Borneans experienced a steady decline in *N*_e_. The *N*_e_ of Bornean orangutans was reduced from ~ 30,000 to ~ 5000 between 300 and 100 ka ago (Fig. 2). Low cross-population *N*_e_ estimates of below 5000 for all population comparisons on Borneo at this time (Fig. 3b) indicate high levels of gene flow among Bornean populations and suggest the possibility of a single common refugium during this period. This was followed by a rapid increase of cross-population *N*_e_ at 20–50 ka, indicating increasing genetic isolation of most populations during the late Pleistocene (Fig. 3b). This result is in good agreement with the momi2 analysis, indicating a split time among Bornean population of ~ 25 ka (23–27 ka; Additional file 1: Table S4). However, the increase in cross-population *N*_e_ was not accompanied by an increase of *N*_e_ within populations, which remained low on Borneo (3000–10,000; Fig. 2, Additional file 1: Table S4). In contrast, on Sumatra, high *N*_e_ during the Middle Pleistocene ended with a rapid decline of about an order of magnitude at ~ 70–80 ka (Fig. 2). Similar to the pattern in Borneo, *N*_e_ in Sumatran orangutans did not recover (Fig. 2) and the site-frequency spectrum-based demographic model resulted in current *N*_e_ estimates as low as 80–230 for the populations on Sumatra (Additional file 1: Table S4).

The divergent demographic histories of the orangutans on Borneo and Sumatra, along with persistent environmental differences on both islands [21, 23, 24], may have had a strong impact on adaptive evolution of this genus, since the efficiency of selection and the adaptive potential depend on *N*_e_ [49]. Considering this, we explored potential genomic adaptions over different evolutionary time scales.

### Selection during more distant time scales

We detected selection over more distant evolutionary time scales based on non-synonymous to synonymous substitution rate ratios within protein-coding genes estimated by Markov codon models (MCM; Additional file 1: SI Section 7). In this highly conservative approach, we applied the branch-site tests for positive selection by Zhang et al. [50] on a set of 34,379 aligned exons of four primate species based on the reference sequences of human, chimpanzee, and rhesus macaque, along with the orangutan samples. We used samples from North Sumatran orangutans (*P*. *abelii*, LK/NA and WA), and Northeast Bornean orangutans (*P*. *pygmaeus morio*, populations SK/NK and EK), ignoring the recently established population structure (Additional file 1: Figure S8).

**Fig. 4:**
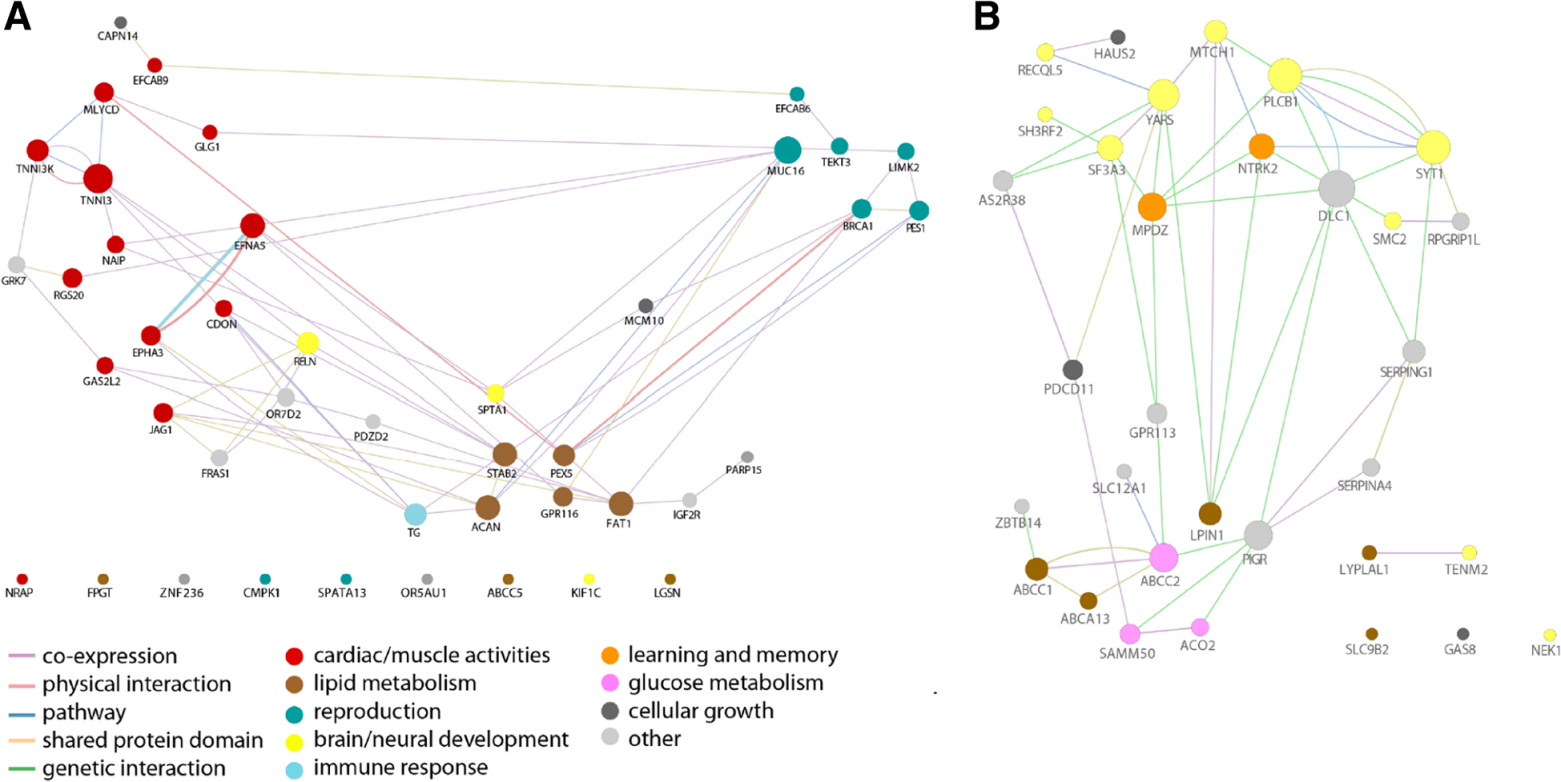
Adaptive history of the genus *Pongo* as inferred from codon modeling (MCM). Interaction networks of all genes under positive selection according to MCM in Northeast Bornean **a** and North Sumatran **b** individuals, generated using GeneMania (v3.4.0) in Cytoscape (v3.3.0) [97]. Each node in the network is a candidate gene identified through the selection test. Genes are labeled with their HUGO Gene IDs. We assigned putative functional classes (fill color) to genes according to the presence of specific keywords (Additional file 1: Table S9) in the GeneCards encyclopedia [51]. Gene interactions are shown by colored lines. The size of gene nodes reflects the number of interaction partners (edges) in the network

Our MCM tests revealed 46 genes with evidence of positive selection in the Northeast Bornean branch and 33 genes in the North Sumatran branch (Additional file 1: Tables S6 and S7). To gain a better understanding of the functions of these genes, we generated interaction networks, where the nodes (genes) were colored based on functional classes according to the presence of specific keywords (Additional file 1: Table S9) in the GeneCards encyclopedia [51] (Fig. 4). The two resulting networks revealed that the functions of the candidate genes differed markedly between the two islands.

Most of the genes under positive selection in Northeast Bornean orangutans were involved either in muscle and cardiac activities, in reproductive pathways, or in the lipid metabolism (Fig. 4a). In line with this observation, gene ontology (GO) enrichment analysis identified 11 GO terms that were significantly enriched for the candidate genes (Table 1), including multiple GO terms involved in fatty acid metabolism and muscle activities, one GO term involved in endocardial cell development, and another in the regulation of fertilization.

**Table 1:** Significantly enriched gene ontology (GO) terms based on MCM

GO term	GO description	*P* FDR^a^	No. of genes^b^
North Sumatra (*P*. *abelii*)			
GO:0005524	ATP binding	1.82 × 10^−4^	9/541
GO:0006200	Obsolete ATP catabolic process	2.47 × 10^−4^	5/125
GO:0007588	Excretion	2.79 × 10^−4^	2/41
GO:0001822	Kidney development	5.49 × 10^−4^	3/83
GO:0048403	Brain-derived neurotrophic factor binding	1.91 × 10^−3^	3/62
GO:0060175	Brain-derived neurotrophic factor-activated receptor activity	1.91 × 10^−3^	3/62
GO:0021987	Cerebral cortex development	3.05 × 10^−3^	3/62
GO:0008331	High voltage-gated calcium channel activity	9.49 × 10^−3^	2/84
Northeast Borneo (*P*. *pygmaeus morio*)			
GO:0051057	Positive regulation of small GTPase-mediated signal transduction	3.13 × 10^−5^	2/35
GO:0051893	Regulation of focal adhesion assembly	1.09 × 10^−4^	2/52
GO:0070507	Regulation of microtubule cytoskeleton organization	1.86 × 10^−4^	3/69
GO:0005540	Hyaluronic acid binding	9.56 × 10^−4^	2/69
GO:0048013	Ephrin receptor signaling pathway	1.87 × 10^−3^	5/121
GO:0000268	Peroxisome targeting sequence binding	2.29 × 10^−3^	4/87
GO:0003025	Regulation of systemic arterial blood pressure	2.29 × 10^−3^	4/87
GO:0050080	Malonyl-CoA decarboxylase activity	2.29 × 10^−3^	4/87
GO:0061444	Endocardial cushion cell development	2.29 × 10^−3^	4/87
GO:0070325	Lipoprotein particle receptor binding	2.29 × 10^−3^	4/87
GO:0080154	Regulation of fertilization	5.72 × 10^−3^	4/104

^a^*P* value after adjustment for multiple testing; ^b^The number of unique genes found for the given GO term related to the total number of genes that could be found at most for this term

Listed GO terms were significantly enriched in the analysis of all protein-coding candidate genes of the MCM selection tests. We only report GO terms related to biological processes

In North Sumatran orangutans, a striking number of positively selected genes were associated with brain and nervous system development as well as energy metabolism pathways (Fig. 4b). Gene ontology enrichment analyses of the these genes (Table 1) revealed a significant enrichment of GO terms related to ATP metabolism, cerebral cortex development, and two GO terms involved in brain-derived neurotrophic factor binding, all of which mediate higher cognitive abilities [52].

### Selection during more recent time scales

To uncover signatures of more recent or ongoing local adaptations, we applied two complementary methods for detecting selective sweeps on the population level (Additional file 1: SI Section 9): (1) haplotype-based statistics (iHS) in windowed whole-genome scans [37], and (2) composite likelihood ratio (CLR) test implemented in SweeD [35]. We split our dataset to account for current population structure [33], subdividing Sumatran samples into the two clusters Northeast Alas (LK/NA) and West Alas (WA), and the Bornean samples into the clusters Northeast Borneo (SK/NK and EK) and Central/West Kalimantan (CK/WK). In order to assess thresholds of significance and minimize the occurrence of false positives due to confounding effects of demography [42], we performed a set of neutral simulations based on a complex demographic model estimated through ABC modeling [33] (Additional file 1: SI Section 9.4). We then used GO enrichment analyses to unravel differences in biological functions of genes found to be under positive selection.

1. Haplotype-based selection test (iHS)

We identified 961 annotated protein-coding genes within genomic candidate regions of positive selection (North Sumatran orangutans: *n* = 605, Bornean orangutans: *n* = 410, both: *n* = 54; Additional file 1: Table S11). Detailed information on all candidate genes, including summaries of their potential function and disease associations in humans and other animals, are provided in Additional file 2: Table S17. In Bornean orangutans, many candidate genes were associated with lipid and glucose metabolism as well as insulin and cholesterol regulation (Additional file 2: Table S18). After correction for multiple testing, GO enrichment analysis revealed one significantly enriched GO term within the Northeast Bornean orangutans which was related to immune system-related cell signaling (Table 2, Additional file 2: Tables S20 and S21).

**Table 2:** Significantly enriched gene ontology (GO) terms based on iHS

GO term	GO description	*P* FDR^a^	No. of genes^b^
Northeast Alas (*P*. *abelii*)			
GO:0050877	Neurological system process	0.0065	31/818
GO:1903433	Regulation of constitutive secretory pathway	0.0178	2/2
GO:0007268	Synaptic transmission	0.0314	14/274
GO:0007612	Learning	0.0383	8/105
GO:0030534	Adult behavior	0.0477	9/135
GO:0044708	Single-organism behavior	0.0492	15/320
West Alas (*P*. *abelii*)			
GO:0042254	Ribosome biogenesis	0.0173	10/110
GO:0021987	Cerebral cortex development	0.0215	9/90
GO:0050919	Negative chemotaxis	0.0367	5/23
GO:0021825	Substrate-dependent cerebral cortex tangential migration	0.0500	3/5
Northeast Borneo (*P*. *pygmaeus morio*)			
GO:2000552	Negative regulation of T-helper 2 cell cytokine production	0.0500	2/2

^a^*P* value after adjustment for multiple testing; ^b^The number of unique genes found for the given GO term related to the total number of genes that could be found at most for this term

Listed GO terms were significantly enriched in the analysis of all protein-coding candidate genes of the iHS selection tests. We only report GO terms related to biological processes

In North Sumatran orangutans, a striking number of positively selected genes were associated with crucial functions in learning and memory (Additional file 2: Table S19). In line with this, GO analyses showed a significant enrichment of terms related to neurological processes, synaptic transmission, learning, adult behavior, and cerebral cortex development (Table 1, Additional file 2: Tables S22 and S23).

(2) Composite likelihood ratio test (SweeD)

We identified 1027 protein-coding genes within genomic candidate regions of positive selection (North Sumatran orangutans: *n* = 658, Bornean orangutans: *n* = 489, both: *n* = 120; Additional file 1: Table S12). After correction for multiple testing, GO enrichment analysis revealed eight significantly enriched GO terms within the Sumatran West Alas population (Table 3). No other orangutan population showed significantly enriched GO terms after correction for multiple testing (Additional file 2: Tables S23–27). Among the significantly enriched GO terms in the West Alas population were such involved in glutamate receptor signaling, regulation of postsynaptic membrane potential, and cell projection organization. Glutamate receptors mediate postsynaptic excitation of neuronal cells and are important for neural communication, memory, and learning [53].

**Table 3:** Significantly enriched gene ontology (GO) terms based on SweeD

GO term	GO description	*P* FDR^a^	No. of genes^b^
West Alas (*P*. *abelii*)			
GO:0007215	Glutamate receptor signaling pathway	0.0004	9/35
GO:0035235	Ionotropic glutamate receptor signaling pathway	0.0045	5/11
GO:0099565	Chemical synaptic transmission, postsynaptic	0.0066	8/44
GO:0060079	Excitatory postsynaptic potential	0.0066	8/43
GO:0060078	Regulation of postsynaptic membrane potential	0.0066	9/58
GO:0061000	Negative regulation of dendritic spine development	0.0175	3/4
GO:0120036	Plasma membrane bounded cell projection organization	0.0184	31/608
GO:0030030	Cell projection organization	0.0227	31/617

^a^*P* value after adjustment for multiple testing; ^b^The number of unique genes found for the given GO term related to the total number of genes that could be found at most for this term

Listed GO terms were significantly enriched in the analysis of all protein-coding candidate genes of the SweeD selection tests. We only report GO terms related to biological processes

## Discussion

Our demographic analyses highlighted the deep divergence of orangutans inhabiting the Southeast Asian islands of Sumatra and Borneo. Despite land bridges occurring between these islands as recently as ~ 10 ka ago [19], we found no evidence of substantial gene flow more recently than 500 ka in our gene flow analyses using either haploid male X chromosomes or entire phased autosomal genomes. This finding is also supported by deep divergence of island-specific mitochondrial haplotypes, even though Y-chromosomal data indicate the presence of low levels of gene flow during the Middle Pleistocene [33].

In contrast, fitting a detailed demographic model to our genome-wide SNP data set, we found evidence for more recent admixture at ~ 44 ka. This recent admixture event falls into the last glacial period (115–12 ka), when sea levels were up to 120 m below the current sea level and the exposed Sunda shelf opened up land bridges in between the islands [54]. However, this recent admixture appears to have affected only small fractions of individual genomes, which might explain why we did not detect it as a drop in cross-population *N*_e_ in the MSMC2 analysis.

A more pervasive genetic exchange over the exposed Sunda shelf might have been prevented by the increasingly drier and seasonal climate of Late Pleistocene glacial periods [24]. A savannah corridor [55], large river systems dissecting the exposed shelf [19], or most likely both might have imposed impassable dispersal barriers for orangutans. This early separation of gene pools had put Sumatran and Bornean orangutans on largely independent evolutionary trajectories, which is apparent from their divergent demographic histories. Most strikingly, while Sumatran orangutans appear to have expanded considerably in *N*_e_ during the Middle Pleistocene, Bornean orangutans experienced a pronounced decline in *N*_e_ following their isolation from the ancestral population.

The Early–Middle Pleistocene transition 1.2–0.5 Ma was characterized by a fundamental change in the Earth’s climate [56], resulting in harsher glacial periods, affecting vegetation patterns in the tropics [57]. In Sundaland, the drier and more seasonal climate during glacial periods seems to have severely impacted Bornean orangutans, probably through contraction of rainforest coverage [24, 58]. Furthermore, El Niño events have likely caused prolonged lean periods in the eastern parts of Borneo [28, 29]. On Sumatra, however, substantial areas are thought to have remained forested during glacial periods, probably facilitated by sustained high levels of precipitation due to convergent rainfall along the Barisan Mountain range [58], which runs along the western side of the entire island. Moreover, the younger geological age of Sumatra compared to Borneo as well as the mineral-rich volcanic soil resulted in higher rainforest productivity [21, 22], potentially alleviating negative impacts of glacial cycles on Sumatran orangutans.

The decline of Bornean orangutans culminated in a very small *N*_e_ during the last glacial period. The combination of small *N*_e_ within populations and high levels of genetic exchange among populations is suggestive of a single refugium on Borneo, possibly linked to a severe contraction of rain forests during the last glacial period [23, 24]. The finding of strong gene flow between Bornean populations until less than 50 ka suggests that the genetic differentiation underlying the phenotypic differences between orangutan populations from central and northeast Borneo have emerged only recently.

In contrast to the long-term negative population dynamics on Borneo, Sumatran orangutans retained a large *N*_e_ until 70–80 ka, when they experienced a severe reduction in *N*_e_. It is notable that this sudden signal coincides with the Toba supereruption ~ 73 ka [59]. The effects of this eruption, however, were probably limited in their geographic extent and likely of relatively short duration [60], as the mineral-rich deposits of Toba provided fertile ground for recovery of the highly productive Sumatran rainforests [22]. Nevertheless, as on Borneo, *N*_e_ did not recover in Sumatran orangutans. It is possible that this reflects hunting by early human colonizers, who expanded rapidly into insular Southeast Asia in the Late Pleistocene [61]; however, other environmental factors may also have been responsible.

The divergent demographic trajectories of orangutans on Borneo and Sumatra, together with persistent environmental differences until the present [22], may have had an important impact on adaptive evolution of this genus, and hence on the biology of these two species [32]. Across a range of selection tests, we found a consistent pattern of between-island (and thus between-species) differences.

In line with environmental constraints on Borneo and frequent episodes of extreme food scarcity [22], selection in Bornean orangutans appears to have manifested itself predominantly in physiological adaptations to the harsher environmental conditions on the island. *P*. *p*. *morio* showed an enrichment of genes under positive selection involved in cardiac activity, whose remodeling is known to enhance efficient usage of restricted energy resources [62]. Adaptive changes in cardiovascular activities have also been shown for others in extreme environments such as for humans in oxygen-deprived high altitudes (reviewed in [63]), polar bears in cold waters [64], and lizards facing seasonal starvation [65]. We also found an enrichment of genes under positive selection involved in lipid metabolism and energy storage. Such metabolic changes might allow for physiological buffering against starvation [25, 28, 66, 67]. This idea is supported by studies of physiology in wild orangutans and observations from captive animals, indicating that Bornean orangutans are better at storing fat in adipose depots than Sumatran orangutans [26, 28, 68]. It is worthwhile to note that in human populations inhabiting tropical rainforest and thus facing limitation and instability in their food supply, genes involved in lipid metabolism and muscle function were also found to be under selection [69].

In contrast to Bornean orangutans, energy budgets of their North Sumatran counterparts are more favorable due to generally higher and more stable food supplies [22]. In agreement with this, and the documented differences in phenotypic traits, we identified a very different set of candidate genes under positive selection, which were mostly linked to the brain and nervous system development, learning and memory processes, and glucose metabolism. It is conceivable that these selective changes have contributed to the development of extended behavioral plasticity via individual and social learning in North Sumatran orangutans, who surpass their Bornean counterparts in the size and complexity of cultural repertoire [32, 70, 71] and the ability to solve cognitive problems [72].

Among the most striking examples of the broad phenotypic variation in the genus *Pongo* are the higher sociability and social tolerance of the North Sumatran orangutans [73–77]. Current population densities are higher on northern Sumatra than on Borneo [20], a situation which may have existed throughout most of the Pleistocene, given the higher estimates of historical *N*_e_ in *P*. *abelii*. Such higher densities may have provided more opportunities for social learning in North Sumatran orangutans, which in conjunction with selection on genes underlying cognitive abilities and prosocial behavior then facilitated the emergence and persistence of larger cultural repertoires compared to their Bornean counterparts. While not empirically evident, our results nevertheless suggest an intriguing link between the demographic history during the Pleistocene glaciations and the cognitive and cultural evolution in orangutans.

## Methods

### Sampling and data generation

Our sample set included whole-genome data from 35 orangutans, representing the entire geographic range of Bornean and North Sumatran orangutans (Additional file 1: Figure S1, Additional file 1: Table S1). All individuals were wild-born, except for five orangutans which were first-generation offspring of wild-born parents of the same species (Additional file 1: Table S2). We obtained whole-genome sequencing data for the study individuals from three different sources (Additional file 1: Tables S1 and S2): (i) Nater et al. [33] (*n* = 16, effective quality-filtered sequencing coverage: 13.7–31.1x), (ii) Prado-Martinez et al. [78] (*n* = 10, 20.5–27.4x), and (iii) Locke et al. [79] (*n* = 9, 4.8–12.2x).

### Read mapping, variant calling, and phasing

We followed identical bioinformatics procedures for all 35 study individuals. Read mapping and variant calling is described in Nater et al. [33] and briefly summarized in Additional file 1: SI Section 2.

### Haplotype phasing

We phased genotype data from *P*. *abelii* and *P*. *pygmaeus* employing SHAPEIT v2.0 [80]. We used a stringent high-quality subset of genotype data from the original SNP-calling dataset for each species, for which we extracted only biallelic and polymorphic SNPs without missing genotype data. We ran the algorithm at chromosome level to generate a haplotype graph, which we used to assess phasing uncertainty and to extract the most likely haplotypes per individual.

### Neighbor-net

We visualized the genetic relationships among the whole-genome sequences by building a neighbor-net using SplitsTree v4.14.6 [81]. To obtain the underlying distance matrix, we calculated pairwise identity-by-state proportions with PLINK v1.90 [82] using only biallelic SNPs with no missing genotypes across all 35 individuals.

### PSMC and MSMC

We inferred orangutan population size history with the pairwise sequentially Markovian coalescent (PSMC) model [43]. PSMC is implemented as a hidden Markov model and allows inferring historical changes in *N*_e_ using the distribution of pairwise coalescent times within a single diploid genome. We applied the PSMC model to each sample, grouping individuals by sequencing coverage to avoid coverage-related biases (Fig. 2a, Additional file 1: Figures S1-S3). We ran PSMC with the parameter settings that were found to be suitable for great apes and have been applied to orangutans previously [43, 78]. We scaled the resulting plots using an autosomal mutation rate of 1.5 × 10^−8^ mutations per base pair per generation and a generation time of 25 years [83].

We inferred ancestral gene flow between islands and between populations within islands using the multiple sequentially Markovian coalescent (MSMC2) model [44]. Whereas PSMC uses information from inter-chromosomal genetic differences within a single diploid genome to infer ancestral *N*_e_, MSMC2 extends this approach to multiple haplotypes from different populations. Since the model requires phased data to pair haploid genome sequences across populations, high phasing accuracy is paramount. To avoid issues with phasing uncertainty, we focused solely on male X chromosomes. We scaled the results using an X-chromosomal mutation rate of 1.17 × 10^−8^ mutations per base pair per generation and a generation time of 25 years [83].

### Codon models

To identify protein-coding genes under positive selection, we used likelihood ratio tests (LRTs) based on pairs of nested Markov models of codon substitutions as implemented in PAML v1.3.1 [84]. Selection on the protein level was measured by the *ω*-ratio of non-synonymous to synonymous substitution rates [85], where higher *ω*-ratio indicates recurrent positive selection.

We used a single consensus FASTA genome sequence with randomly resolved haplotype phase for each individual. We identified the genomic positions of exons by retrieving a list of all unique protein-coding exons with Ensembl transcript IDs for the orangutan reference genome *ponAbe2* from the UCSC Table Browser [86], only considering exons that (i) were at least 60 nucleotides long, (ii) had a number of nucleotides with multiples of 3, and (iii) contained at least one non-synonymous substitution in one of the individuals. As outgroups, we used human (hg18), chimpanzee (panTro4), and rhesus macaque (rheMac3) reference genomes from the UCSC Genome Browser [87]. Multiple sequence alignments for each exon were constructed using the multiple alignment format files from the UCSC Genome Browser, applying AlignIO and MafIO tools of Biopython v1.60+ [88] with default parameters.

Gene trees were inferred separately for each protein-coding exon based on codon model M0 with one ω-ratio per gene as implemented in CodonPhyML v1.0. [89] using the initial species topology in Additional file 1: Figure S14. We applied a test for variability of *ω* among sites (comparing models M0 vs. M3). For exons with significant variation of *ω* in their sequences, we fitted the nested site models (M8 vs. M8a) to identify exons with evidence for positive selection. For this subset of exons, we then performed a LRT for positive selection based on branch-site models, where we compared the modified model A to the corresponding null model with *ω* fixed to 1 at the “foreground” branches. Here, we defined either the North Sumatran or the Northeast Bornean (all *P*. *pygmaeus morio* individuals) lineages as foreground.

### Genome scans

To uncover signatures of recent or ongoing selective sweeps, we applied two complementary tests of selection in genome-wide windows: (1) a haplotype-based selection test to identify extended linkage disequilibrium patterns surrounding recently selected alleles (iHS) [36, 37] and (2) a composite likelihood ratio (CLR) test to detect shifts in local site-frequency spectra relative to the genomic background. We used the R package *rEHH* [90] with default parameters to obtain absolute iHS scores for each variant position in each population and averaged iHS scores in sliding 25-kb windows with 12.5 kb step size. To calculate the CLR test statistic, we used the program SweeD v3.3.2 [35], applying a spacing of grid points of 12.5 kb and using the unfolded site-frequency spectrum. To prepare the input files, we used the phased genotype data described above and additionally determined the ancestral state for each SNP by comparing the alleles at each variant position with human and chimpanzee outgroup genotypes. We excluded sites that were not biallelic, for which the ancestral state could not be determined, or which were monomorphic at the population level. To identify significant departure from patterns expected under neutrality, we ran coalescent simulations under a detailed demographic model estimated earlier [33], generating 10,000 replicate sequences of 100 kb for comparison with the empirical data.

### Gene ontology enrichment analysis

For the MCM analyses, we retrieved gene ontology (GO) terms associated with the positively selected orangutan genes using the BioMart data mining tool from Ensembl v82 [91]. We identified significantly enriched GO terms by computing the *χ*^2^ statistic for each GO term in a given set of positively selected genes per analysis against the set of all genes considered in that analysis, applying FDR multiple testing correction [92]. For the iHS and SweeD analyses, we performed GO enrichment analyses using the R package “gProfileR” of the g:Profiler toolkit [93, 94]. Significance was assessed by comparing the candidate genes with a background list of all possible genes, i.e., all protein-coding genes (*n* = 12,866) located within any window with sufficient coverage for calculation of the iHS and CLR statistics. We applied the Benjamini-Hochberg method [92] for computing multiple testing correction for *P* values gained from GO enrichment analysis.

Detailed methods and materials are provided in Additional file 1: Supplementary Information.

## Conclusions

We describe the interplay between environmental processes, demography, and adaptive evolution in a non-human great ape, the orangutan. We found that in the relatively stable environment of Sumatra, selection acted primarily on genes linked to brain development and learning, consistent with well-documented cognitive and cultural differences between species*.* On Borneo, however, selection manifested itself in physiological adaptations to harsher and more fluctuating environmental conditions. These findings may have important implications for the study of hominid evolution, as they suggest a link between cognitive and cultural evolution in great apes and the presence of high habitat productivity and demographic stability. Thus, our study provides a framework from which to develop and test more complex hypotheses about adaptive evolution in non-human great apes and to explore differences in adaptive evolution between our own species and our closest relatives.

## Additional files

Additional file 1: Supplementary Information. (PDF 1813 kb)

Additional file 2: **Table S13.** List of SNPs completely fixed for different alleles in Bornean and North Sumatran orangutans. **Table S14.** Functional consequences of SNPs completely fixed between North Sumatran and Bornean orangutans. **Table S15.** Results of the GO enrichment analysis of fixed SNPs. **Table S16.** Detailed information on all genes containing fixed non-synonymous SNPs between North Sumatran and Bornean orangutans. **Table S17.** Detailed information on all candidate genes of the iHS analysis. **Table S18.** Selection of candidate genes of the iHS analysis in Bornean orangutans. **Table S19.** Selection of candidate genes of the iHS analysis in North Sumatran orangutans. **Table S20.** Results of GO enrichment analysis of SK/NK/EK for the iHS selection test. **Table S21.** Results of GO enrichment analysis of CK/WK for the iHS selection test. **Table S22.** Results of GO enrichment analysis of LK/NA for the iHS selection test. **Table S23.** Results of GO enrichment analysis of WA for the iHS selection test. **Table S24.** Results of GO enrichment analysis of CK/WK for the SweeD selection test. **Table S25.** Results of GO enrichment analysis of SK/NK/EK for the SweeD selection test. **Table S26.** Results of GO enrichment analysis of LK/NA for the SweeD selection test. **Table S27.** Results of GO enrichment analysis of WA for the SweeD selection test. (XLSX 13976 kb)
