# Supplementary material for: Genomes reveal marked differences in the adaptive evolution between orangutan species
Source: Genome Biol. 2018 Nov 15;19:193. doi: 10.1186/s13059-018-1562-6 (PMC6237011; doi:10.1186/s13059-018-1562-6)
Supplement: Supplementary file 1 — Supplementary Information. (PDF 1813 kb) [file 13059_2018_1562_MOESM1_ESM.pdf]

Additional file 1

SUPPLEMENTARY INFORMATION

Genomes reveal marked differences in the adaptive evolution between orangutan species

Maja P. Mattle-Greminger[§], Tugce Bilgin Sonay[§], Alexander Nater[§], Marc Pybus, Tariq Desai, Guillem de Valles, Ferran Casals, Aylwyn Scally, Jaume Bertranpetit, Tomas Marques-Bonet, Carel P. van Schaik, Maria Anisimova, and Michael Krützen

[§]These authors contributed equally to this work.

Table of Contents

Section 1: Sample collection 3

Section 2: Whole-genome sequencing, read mapping, SNP, genotype calling, and haplotype phasing 5

Section 3: PSMC analysis 9

3.1 Methods 9

3.2 Results 9

Section 4: Ancestral gene flow between orangutan populations 12

4.1 Methods 12

4.2 Results 13

Section 5: Mutational load analysis 20

Section 6: Fixed SNPs between species 23

6.1 Methods 23

6.2 Results 23

Section 7: Codon models for positive selection analysis 26

Section 8: Interaction network analyses 30

Section 9: Population-based selection analyses 31

9.1 Methodological considerations 31

9.2 Data sets for population-level analyses 31

9.3 Tests for positive selection 32

9.4 Neutral simulations 32

9.5 Candidate gene information and GO enrichment analysis 33

9.6 Overlap of candidate genes among populations 33

References 35

Section 1: Sample collection

We used whole-genome sequencing data from 35 orangutans, covering the entire geographic range of North Sumatran (*Pongo abelii*) and Bornean (*P. pygmaeus*) orangutans (Fig. 1, Tables S1 and S2). Data from 16 individuals was obtained from Nater et al. [1], genomes of 10 orangutans were sequenced in Prado-Martinez et al. [2], and data for 9 individuals were taken from Locke et al. [3]. Most individuals were wild-born, except for five orangutans which were first-generation offspring of wild-born parents of the same species (Table S2).

Table S1. Overview of orangutan samples. Effective read-depths are given as ranges in brackets below the source reference. Numbers in parentheses are individuals born in captivity.

**Species**	**Sampling areas**	**Nater et al. 2017** [13.7-31.1x]	**Prado-Martinez et al. 2013** [20.5-27.4x]	**Locke et al. 2011** [4.8-12.2x]	**Total**
P. abelii	Langkat (LK)	0	4	2	6
P. abelii	North Aceh (NA)	1	(1)	0	1+(1)
P. abelii	West Alas (WA)	5	0	2	7
P. pygmaeus morio	South Kinabatangan (SK)	2	0	0	2
P. pygmaeus morio	North Kinabatangan (NK)	2	0	0	2
P. pygmaeus morio	East Kalimantan (EK)	2	0	1	3
P. pygmaeus pygmaeus	Sarawak (SR)	1+(1)	1	1	3+(1)
P. pygmaeus wurmbii	Central Kalimantan (CK)	(1)	2+(2)	3	5+(3)
P. pygmaeus wurmbii	West Kalimantan (WK)	1	0	0	1

Table S2. Details of study individuals.

Species	Sampling area	Individual ID	Individual name	Sex	Mean depth[a]	Source	Comments and origin details, if available
P. abelii	Langkat	PA_KB4661	Bubbles	M	4.76	Locke et al. [3]	Wild-born
P. abelii	Langkat	PA_KB5883	Sibu	M	4.99	Locke et al. [3]	Wild-born
P. abelii	Langkat	PA_A947	Elsi	F	27.39	Prado-Martinez et al. [2]	Wild-born
P. abelii	Langkat	PA_A948	Kiki	F	23.71	Prado-Martinez et al. [2]	Wild-born
P. abelii	Langkat	PA_A950	Babu	F	26.28	Prado-Martinez et al. [2]	Wild-born
P. abelii	Langkat	PA_A952	Buschi	M	21.03	Prado-Martinez et al. [2]	Wild-born
P. abelii	North Aceh	PA_A949	Dunja	F	27.39	Prado-Martinez et al. [2]	1st Generation by 456 and 457 both wild-born Sumatra
P. abelii	North Aceh	PA_B018	Jeff	M	16.31	Nater et al. [1]	Wild-born; Desa Seuneubok Bayu, Kec. Indra Makmu
P. abelii	West Alas	PA_KB4361	Likoe	F	5.66	Locke et al. [3]	Wild-born
P. abelii	West Alas	PA_SB550	Doris	F	4.86	Locke et al. [3]	Wild-born
P. abelii	West Alas	PA_B017	Miky	F	13.74	Nater et al. [1]	Wild-born; Aluebillie, Aceh Nagan Raya, Aceh province
P. abelii	West Alas	PA_A953	Vicky	F	17.78	Nater et al. [1]	Wild-born
P. abelii	West Alas	PA_A955	Suma	F	25.27	Nater et al. [1]	Wild-born
P. abelii	West Alas	PA_A964	Rochelle	F	11.06	Nater et al. [1]	Wild-born
P. abelii	West Alas	PA_B020	Maini	F	16.3	Nater et al. [1]	Wild-born; Aceh Sealatan near Suaq Balimbing
P. pygmaeus	Central Kalimantan	PP_KB4204	Dolly	M	5.61	Locke et al. [3]	Wild-born
P. pygmaeus	Central Kalimantan	PP_KB5404	Billy	F	12.24	Locke et al. [3]	Wild-born
P. pygmaeus	Central Kalimantan	PP_KB5405	Dennis	M	5.61	Locke et al. [3]	Wild-born
P. pygmaeus	Central Kalimantan	PP_A940	Temmy	F	21.8	Prado-Martinez et al. [2]	1st Generation by 793 and 794 both wild-born Borneo
P. pygmaeus	Central Kalimantan	PP_A941	Sari	F	23.17	Prado-Martinez et al. [2]	1. Gen. by 202 and 322 both wild-born Borneo
P. pygmaeus	Central Kalimantan	PP_A943	Tilda	F	24.17	Prado-Martinez et al. [2]	Wild-born
P. pygmaeus	Central Kalimantan	PP_A944	Napoleon	M	23.32	Prado-Martinez et al. [2]	Wild-born
P. pygmaeus	Central Kalimantan	PP_A938	Lotti	F	18.62	Nater et al. [1]	1st Generation by 358 and 422 both wild-born Borneo
P. pygmaeus	West Kalimantan	PP_A983	Claus	M	29.71	Nater et al. [1]	Wild-born; Pontianak
P. pygmaeus	East Kalimantan	PP_KB5543	Louis	M	6.03	Locke et al. [3]	Wild-born
P. pygmaeus	East Kalimantan	PP_A984	Barong	F	29.89	Nater et al. [1]	Wild-born; Taman Nasional Kutai
P. pygmaeus	East Kalimantan	PP_A985	Panjul	M	30.13	Nater et al. [1]	Wild-born; Taman Nasional Kutai
P. pygmaeus	North Kinabatangan	PP_A987	Tara	F	30.65	Nater et al. [1]	Wild-born; Bukit Garam, Kinabatangan area
P. pygmaeus	North Kinabatangan	PP_A988	Kala	M	31.06	Nater et al. [1]	Wild-born; Kg. Tikolod, Tambunan
P. pygmaeus	South Kinabatangan	PP_5062	Ampal	M	13.81	Nater et al. [1]	Wild-born; Lahad Datu, Kinabatangan area
P. pygmaeus	South Kinabatangan	PP_A989	Micelle	F	27.30	Nater et al. [1]	Wild-born; Lahad Datu, Kinabatangan area
P. pygmaeus	Sarawak	PP_KB5406	Dinah	F	4.90	Locke et al. [3]	Wild-born
P. pygmaeus	Sarawak	PP_A939	Nonja	F	20.48	Prado-Martinez et al. [2]	1st Generation by 1052 and 1012 both from Sarawak
P. pygmaeus	Sarawak	PP_A942	Gusti	F	23.12	Nater et al. [1]	1st Generation by 1435 and 1392 both wild-born Borneo
P. pygmaeus	Sarawak	PP_A946	Kajan	M	22.39	Nater et al. [1]	Wild-born

[a]mean effective whole-genome sequencing coverage. We estimated sequence depth on the filtered BAM files where duplicated reads, bad read mates, reads with mapping quality zero, and reads which mapped ambiguously had already been removed. Thus, our sequence coverage estimates correspond to the effective read-depths which are available for SNP discovery and genotyping (see Nater et al. [1]).

Section 2: Whole-genome sequencing, read mapping, SNP, genotype calling, and haplotype phasing

We followed identical bioinformatics procedures for all 35 study individuals. Read mapping and variant calling is described in Nater et al. [1]. Basic sequencing and mapping statistics of all 35 study individuals are provided in the Tables S2 and S3. Briefly, we mapped reads to the *ponAbe2* reference genome [3] using BWA-MEM v0.7.5 [4] and removed duplicate reads with Picard v1.101 (http://picard.sourceforge.net/). We performed local realignment around indels and empirical base quality score recalibration (BQSR) with the Genome Analysis Toolkit (GATK) v3.2.2. [5, 6]. We used the *HaplotypeCaller* of the GATK to obtain individual genotype likelihoods and genotyped individuals on a per-island level, as well as combined for all individuals, using the *Genotype GVCFs* tool of the GATK. To identify high-confidence SNPs, we performed variant quality score recalibration (VQSR) on the candidate SNP files, using a 'true SNP' set containing 5,600 high-confidence SNPs, which were independently identified by three different variant callers in a previous reduced-representation sequencing project [7].

We also produced high-quality genomic consensus FASTA sequences for each study individual. We used custom Perl scripts to create the consensus sequences by merging the information of the SNP VCF and gVCF files as following: all sites (variant and reference sites) had to be covered by at least eight individuals per island or genotypes in all individuals at this site were set to 'N'. SNP and genotype calling at genomic positions covered by fewer individuals is less accurate, hence the power to discriminate between variant and non-variant genomic positions is reduced. In addition, we filtered out sites with a mean mapping quality below 20. On the individual level, we required genotypes of both variant and reference positions to be covered by at least three reads, otherwise individual genotypes at that site were set to 'N'. Positions not sequenced for a given individual were also denoted as 'N'. The sequence depth of all non-variant sites in the reference genome (*i.e.*, reference sites) for each individual was obtained from the gVCF files (also mappability masked) produced in the first step of the SNP calling pipeline. The genotype at variant genomic sites was extracted for each individual from the SNP VCF file described above. Heterozygous genotypes were encoded with their respective IUPAC codes.

Genotype data from *P. abelii* and *P. pygmaeus* were phased using the SHAPEIT v2.0 [8] as described in Nater et al. [1]. A high-quality subset of genotype data from the original SNP-calling dataset was used for each species, for which only biallelic and polymorphic SNPs without missing genotype data were extracted. SHAPEIT was run using the following parameters: 100 states, a window size of 0.5 Mb, and two species-specific recombination maps, which had been estimated using LDhat. The algorithm was run at chromosome level to generate a haplotype graph, which was used to assess phasing uncertainty and to extract the most likely haplotypes per individual.

To infer the ancestral states of variants segregating within *Pongo*, we repeated the mapping and genotype calling procedure with whole-genome sequencing data from two individuals each of human (SRA sample accession: ERS007255 and ERS007266) and common chimpanzee (*Pan troglodytes*, SRA sample accession: SRS394801 and SRS396844). All short reads from these outgroup individuals were mapped against the *ponAbe2* reference genome and genotypes were called on a per-species basis with GATK *UnifiedGenotyper* using the set of high-confidence SNP sites identified in *Pongo*. We then applied a parsimony approach to assign ancestral states to SNPs and recoded the *Pongo* genotype

VCF files in order to represent the ancestral state as the reference allele. We did not assign ancestral states for variants for which i) data were missing in both outgroup species, ii) data were missing in one outgroup species and the other species was polymorphic, iii) both outgroup species were polymorphic, iv) both outgroup species were monomorphic for different alleles, or v) three or more alleles were present over both outgroup species. In such cases, we coded all genotypes for this site in the recoded *Pongo* VCF files as missing.

Table S3. Basic sequencing and mapping statistics of orangutan whole-genome sequencing data.

Species	Individual ID	Source	Total no. of reads	No. of reads filtered	% reads filtered	No. of bad mate reads[a]	% bad mate reads
P. abelii	PA_A947	Prado-Martinez et al. [2]	1,199,070,495	217,651,201	18.15%	31,965,745	2.67%
P. abelii	PA_A948	Prado-Martinez et al. [2]	1,026,568,611	212,620,172	20.71%	23,791,092	2.32%
P. abelii	PA_A949	Prado-Martinez et al. [2]	1,238,435,940	295,572,494	23.87%	28,597,946	2.31%
P. abelii	PA_A950	Prado-Martinez et al. [2]	1,221,075,045	277,425,024	22.72%	26,033,305	2.13%
P. abelii	PA_A952	Prado-Martinez et al. [2]	1,061,059,740	333,654,395	31.45%	26,183,487	2.47%
P. abelii	PA_A953	Nater et al. [1]	863,795,942	240,805,194	27.88%	16,835,303	1.95%
P. abelii	PA_A955	Nater et al. [1]	1,151,082,160	258,616,853	22.47%	25,494,067	2.21%
P. abelii	PA_A964	Nater et al. [1]	1,118,829,477	739,560,932	66.10%	18,622,494	1.66%
P. abelii	PA_B017	Nater et al. [1]	1,114,451,019	576,916,768	51.77%	320,927,625	28.80%
P. abelii	PA_B018	Nater et al. [1]	1,213,126,904	606,523,688	50.00%	380,058,442	31.33%
P. abelii	PA_B020	Nater et al. [1]	1,063,963,834	467,186,672	43.91%	268,296,390	25.22%
P. abelii	PA_KB4361	Locke et al. [3]	502,515,251	102,527,136	20.40%	5,668,056	1.13%
P. abelii	PA_KB4661	Locke et al. [3]	395,184,293	76,284,313	19.30%	4,802,843	1.22%
P. abelii	PA_KB5883	Locke et al. [3]	470,563,961	115,172,006	24.48%	7,246,997	1.54%
P. abelii	PA_SB550	Locke et al. [3]	420,906,050	87,518,248	20.79%	6,816,380	1.62%
P. pygmaeus	PP_5062	Nater et al. [1]	520,463,882	71,616,442	13.76%	12,238,321	2.35%
P. pygmaeus	PP_A938	Nater et al. [1]	878,679,380	219,613,306	24.99%	18,771,601	2.14%
P. pygmaeus	PP_A939	Prado-Martinez et al. [2]	982,875,157	258,243,405	26.27%	22,266,716	2.27%
P. pygmaeus	PP_A940	Prado-Martinez et al. [2]	879,365,509	111,712,294	12.70%	23,951,485	2.72%
P. pygmaeus	PP_A941	Prado-Martinez et al. [2]	974,172,871	162,961,808	16.73%	22,324,151	2.29%
P. pygmaeus	PP_A942	Nater et al. [1]	1,119,665,510	294,172,924	26.27%	27,378,538	2.45%
P. pygmaeus	PP_A943	Prado-Martinez et al. [2]	1,137,225,178	276,513,275	24.31%	28,140,416	2.47%
P. pygmaeus	PP_A944	Prado-Martinez et al. [2]	1,110,367,688	280,618,436	25.27%	30,240,109	2.72%
P. pygmaeus	PP_A946	Nater et al. [1]	944,435,510	165,822,299	17.56%	19,510,440	2.07%
P. pygmaeus	PP_A983	Nater et al. [1]	1,150,227,749	171,282,032	14.89%	27,964,164	2.43%
P. pygmaeus	PP_A984	Nater et al. [1]	1,166,011,497	181,228,288	15.54%	32,704,080	2.80%
P. pygmaeus	PP_A985	Nater et al. [1]	1,188,314,591	190,300,804	16.01%	38,933,010	3.28%
P. pygmaeus	PP_A987	Nater et al. [1]	1,182,067,514	169,028,331	14.30%	32,242,622	2.73%
P. pygmaeus	PP_A988	Nater et al. [1]	1,184,387,913	159,637,530	13.48%	28,471,644	2.40%
P. pygmaeus	PP_A989	Nater et al. [1]	1,182,468,671	254,009,220	21.48%	111,433,632	9.42%
P. pygmaeus	PP_KB4204	Locke et al. [3]	488,513,841	91,445,743	18.72%	5,431,871	1.11%
P. pygmaeus	PP_KB5404	Locke et al. [3]	1,223,090,264	279,931,929	22.89%	26,711,713	2.18%
P. pygmaeus	PP_KB5405	Locke et al. [3]	450,850,553	102,845,293	22.81%	3,703,627	0.82%
P. pygmaeus	PP_KB5406	Locke et al. [3]	427,501,183	79,470,592	18.59%	5,199,834	1.22%
P. pygmaeus	PP_KB5543	Locke et al. [3]	531,449,862	133,019,779	25.03%	7,881,803	1.48%

Table S3 (Continued)

Species	Individual ID	No. of duplicate reads	% duplicate reads	No. of MappingQualityZero reads	% MappingQualityZero reads	No. of NotPrimaryAlignment reads[b]	% NotPrimaryAlignment reads
P. abelii	PA_A947	101,285,592	8.45%	83,954,851	7.00%	445,013	0.04%
P. abelii	PA_A948	110,628,113	10.78%	77,752,127	7.57%	448,840	0.04%
P. abelii	PA_A949	185,752,688	15.00%	80,721,416	6.52%	500,444	0.04%
P. abelii	PA_A950	121,393,826	9.94%	129,564,956	10.61%	432,937	0.04%
P. abelii	PA_A952	235,278,445	22.17%	71,714,221	6.76%	478,242	0.05%
P. abelii	PA_A953	165,539,909	19.16%	58,112,480	6.73%	317,502	0.04%
P. abelii	PA_A955	157,826,898	13.71%	74,849,426	6.50%	446,462	0.04%
P. abelii	PA_A964	650,727,730	58.16%	69,762,441	6.24%	448,267	0.04%
P. abelii	PA_B017	19,106,249	1.71%	214,074,036	19.21%	22,808,858	2.05%
P. abelii	PA_B018	14,010,011	1.15%	186,627,308	15.38%	25,827,927	2.13%
P. abelii	PA_B020	16,041,711	1.51%	165,324,329	15.54%	17,524,242	1.65%
P. abelii	PA_KB4361	26,047,292	5.18%	70,810,607	14.09%	1,181	0.00%
P. abelii	PA_KB4661	14,107,709	3.57%	57,371,308	14.52%	2,453	0.00%
P. abelii	PA_KB5883	39,113,310	8.31%	68,810,275	14.62%	1,424	0.00%
P. abelii	PA_SB550	15,906,300	3.78%	64,792,367	15.39%	3,201	0.00%
P. pygmaeus	PP_5062	7,891,952	1.52%	51,019,044	9.80%	467,125	0.09%
P. pygmaeus	PP_A938	143,064,905	16.28%	57,302,521	6.52%	474,279	0.05%
P. pygmaeus	PP_A939	166,225,495	16.91%	69,332,393	7.05%	418,801	0.04%
P. pygmaeus	PP_A940	21,089,546	2.40%	66,213,038	7.53%	458,225	0.05%
P. pygmaeus	PP_A941	75,201,269	7.72%	65,002,932	6.67%	433,456	0.04%
P. pygmaeus	PP_A942	186,368,059	16.64%	79,902,965	7.14%	523,362	0.05%
P. pygmaeus	PP_A943	166,560,691	14.65%	81,341,070	7.15%	471,098	0.04%
P. pygmaeus	PP_A944	163,330,781	14.71%	86,536,463	7.79%	511,083	0.05%
P. pygmaeus	PP_A946	67,691,610	7.17%	78,230,815	8.28%	389,434	0.04%
P. pygmaeus	PP_A983	12,085,031	1.05%	130,143,364	11.31%	1,089,473	0.09%
P. pygmaeus	PP_A984	13,694,397	1.17%	133,233,733	11.43%	1,596,078	0.14%
P. pygmaeus	PP_A985	10,739,096	0.90%	139,051,397	11.70%	1,577,301	0.13%
P. pygmaeus	PP_A987	12,414,888	1.05%	123,045,423	10.41%	1,325,398	0.11%
P. pygmaeus	PP_A988	12,024,936	1.02%	117,985,391	9.96%	1,155,559	0.10%
P. pygmaeus	PP_A989	12,366,091	1.05%	123,005,953	10.40%	7,203,544	0.61%
P. pygmaeus	PP_KB4204	7,718,787	1.58%	78,293,575	16.03%	1,510	0.00%
P. pygmaeus	PP_KB5404	25,789,966	2.11%	227,395,875	18.59%	34,375	0.00%
P. pygmaeus	PP_KB5405	35,353,666	7.84%	63,732,088	14.14%	55,912	0.01%
P. pygmaeus	PP_KB5406	12,921,773	3.02%	61,347,330	14.35%	1,655	0.00%
P. pygmaeus	PP_KB5543	52,266,887	9.83%	72,854,176	13.71%	16,913	0.00%

[a], reads whose mate mapped to a different contig; [b], reads with non-unique mapping.

Section 3: PSMC analysis

3.1 Methods

We inferred orangutan population size history with the pairwise sequentially Markovian coalescent (PSMC) model [9], which uses single diploid genome sequences to reconstruct population size changes through time. The PSMC is implemented as a hidden Markov model in which the observed states correspond to the sequence of homozygous and heterozygous genotypes along the genome. The hidden state is the coalescent time of the two chromosomes at a given position, and transitions between hidden states represent ancestral recombination events. Thus, the PSMC model allows estimating historical changes in N_e based on the distribution of the time to the most recent common ancestor (TMRCA) for alleles within a diploid genome.

We applied the PSMC model to each sample. Input files for PSMC were created from the autosomal consensus FASTA sequences described above, using the utility 'fq2psmcfa' (provided with the PSMC package). We ran PSMC with the following parameter settings, which were found to be suitable for great apes and applied to orangutans previously [2, 9]: 'psmc -N25 -t15 -r5 -p "4+25*2+4+6" -o output.psmc input.psmcfa'. The parameter '-N' defines the number of iterations, '-t' the maximum TMRCA (in the 2N0 scale), '-r' the ratio of theta over rho, and '-p'describes the temporal binning parameters. In our case there were 64 atomic time intervals and 28 (=1+25+1+1) free interval parameters. We measured the variance of N_e estimates by bootstrapping. For each individual, we split its consensus sequence into 50-Mb segments using the 'splitfa' utility (PSMC package), and randomly sampled with replacement from these segments applying the '-b' option in PSMC for 100 rounds.

PSMC plots were drawn with an in-house modified version of the 'psmc_plot.pl' script of the PSMC package. We scaled results to real time, assuming a generation time of 25 years [10] and a mutation rate of 1.5×10^{-8} per site per generation [1] [11-14]. We generated different plots for high-coverage (≥ 20x), mid-coverage (11–18x), and low-coverage (5–6x) genomes as the trajectories of N_e should only be compared among genomes with similar read-depths. This is because the lower the coverage the higher the risk of missing a true heterozygous genotype, leading to reduced TMRCA in PSMC analyses [9].

3.2 Results

Trajectories of the PSMC suggest that Bornean and North Sumatran orangutans diverged ~0.8–1.1 Ma (scaling 0.6×10^{-9} per base pair per year; Fig. 2 and Fig. S1). Subsequently, the two species experienced very different demographic histories. Bornean orangutans underwent an initial population decline followed by short recovery. Around 300 ka, N_e began to decline continuously, resulting in very low N_e in the more recent past. In contrast, N_e of North Sumatran orangutans increased considerably after species separation, which could represent actual population growth, a signal of increased population sub-structuring, or most likely a combination of both. More recently (50–100 ka) autosomal N_e of North Sumatran orangutans dropped sharply to just a few thousand individuals, coinciding with the Toba supereruption ~73 ka [15]. Within species, the trajectories of N_e were highly similar for individuals across populations as shown by the overlap of the variance of their PSMC estimates (Fig. S1).

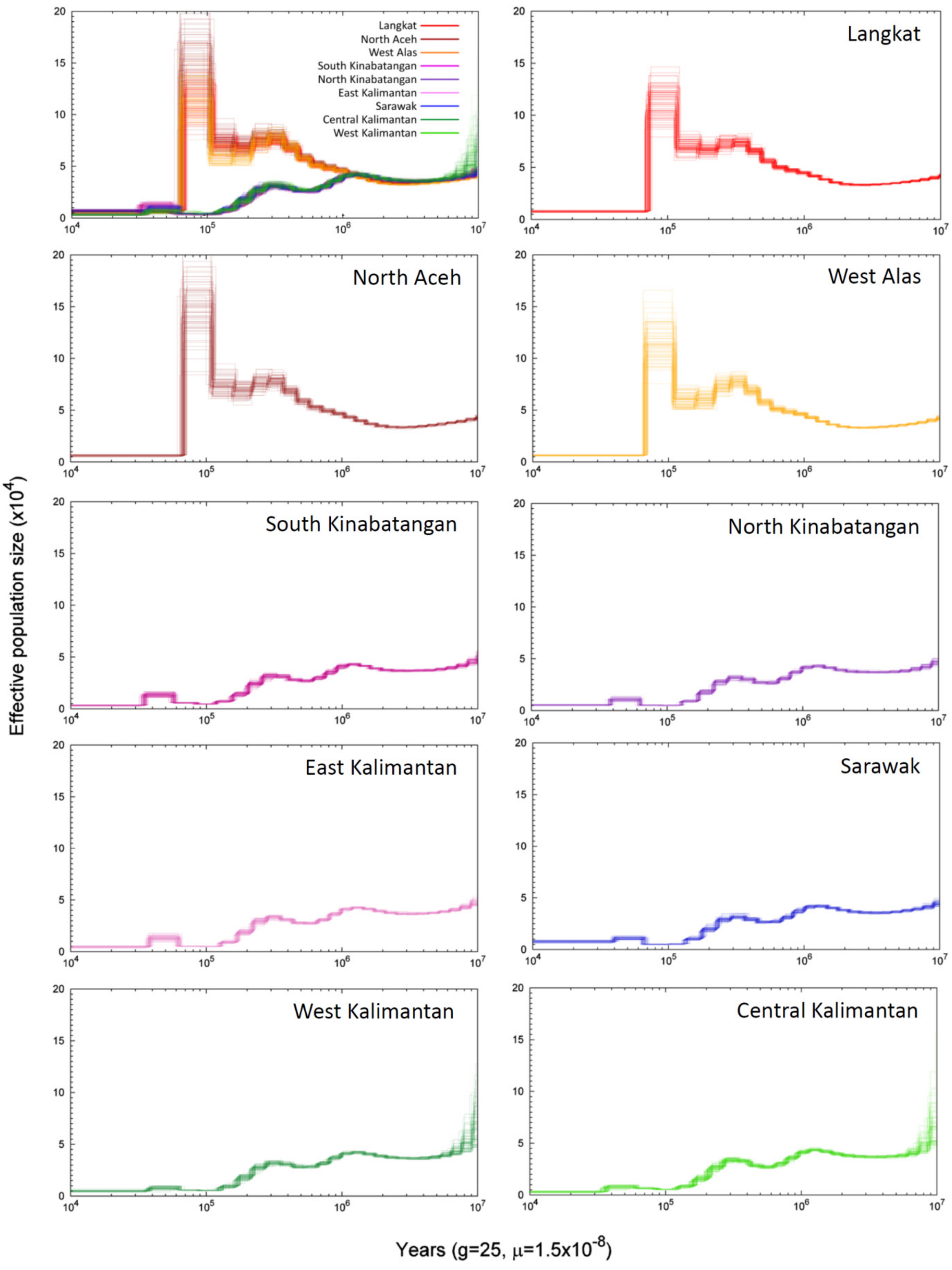

Fig. S1. PSMC bootstrapping plots for individuals in Fig. 2. The x-axis shows time scaled in years, assuming a generation time of 25 years and a mutation rate of $1.5x10^{-8}$ per site per generation. The y-axis shows historical N_e. The fluctuation of the 100 bootstrap replicates indicates the variance. The plot on the top left shows the overlay of all nine individuals. Color codes match those of Fig. 1.

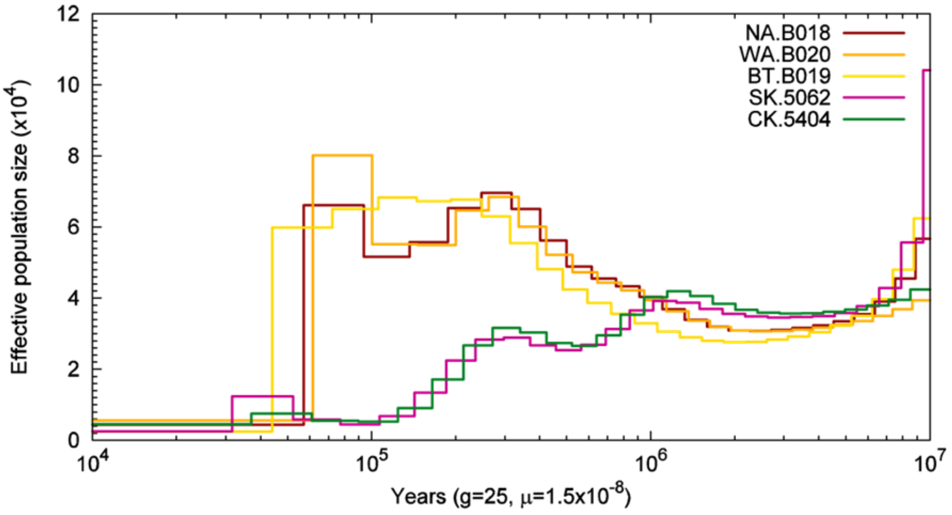

Fig. S2. PSMC analysis of mid-coverage (11–18x) orangutan genomes. The x-axis shows time scaled in years, assuming a generation time of 25 years and a mutation rate of 1.5x10^{-8} per site per generation. The y-axis shows historical N_e. Color codes match those of Fig. 1. Details on individuals can be found in Table S2.

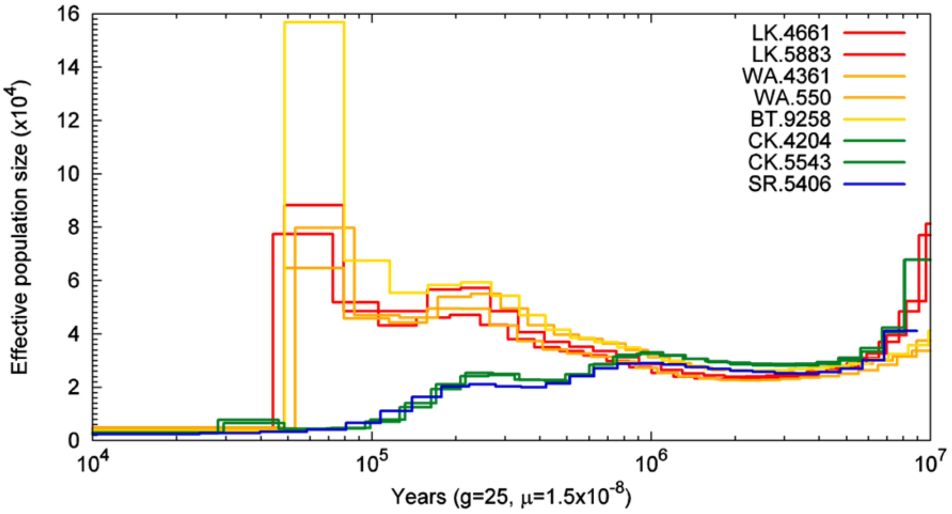

Fig. S3. PSMC analysis of low-coverage (5–6x) orangutan genomes. The x-axis shows time scaled in years, assuming a generation time of 25 years and a mutation rate of 1.5x10^{-8} per site per generation. The y-axis shows historical N_e. Color codes match those of Fig. 1. Details on individuals can be found in Table S2.

Section 4: Ancestral gene flow between orangutan populations

4.1 Methods

Inference of ancestral gene flow was carried out using a method based on the pairwise sequentially Markovian coalescent (PSMC) model [9], namely the multiple sequentially Markovian coalescent (MSMC2) model (https://github.com/stschiff/msmc2). Whereas PSMC uses information from inter-chromosomal genetic differences within a single diploid genome to infer ancestral N_e, MSMC2 extends this approach to multiple genomes (or more than two haplotypes). When run on a pair of haplotypes, the core method in MSMC2 reduces to PSMC. On more than two haplotypes it systematically averages the pairwise analysis.

Following a strategy used by Li & Durbin [9], we exploited the method to explore temporal patterns of gene flow between two populations. This was done by treating a pair of haplotypes, one from each population, as a pseudo-diploid sequence on which the programs were run. To avoid issues with phasing uncertainty, we focused on male X chromosomes, but additionally validated the results with phased autosomal genomes. This approach to analyze gene flow between populations can be justified by considering two populations which diverged at some time T in the past with no subsequent gene flow. We would expect no loci in the pseudo-diploid genome to coalesce more recently than T, and both PSMC and MSMC2 would infer an effectively infinite N_e between T and the present. Prior to T, the inferred N_e should match that obtained in an analysis of the (diploid) X chromosome of a female individual from either population.

Complex demographic histories introduce uncertainty into the estimates of divergence times. This might happen if populations, for example, diverged gradually in the presence of ongoing gene flow. Nevertheless, the inverse of the inferred N_e indicates the extent of genetic exchange between two populations following the split.

To avoid issues with low-coverage genomes, we excluded the following individuals from the analysis: PA_KB5883 and PA_KB4661 from the North Aceh population, PP_KB4204 and PP_KB5405 from Central/West Kalimantan, and PP_KB5543 from East Kalimantan. For all remaining male individuals, we prepared input files in accordance with the requirements specified for MSMC2 (available at https://github.com/stschiff/msmc), by using Python scripts based on the conversion tool generate_multihetsep.py found at https://github.com/stschiff/msmc-tools. Based on the mappability mask described above, we minimized spurious variant calls when generating the input files. Default time discretization parameters were used throughout, but we note that reasonable modifications to them did not substantially affect our results. Cross-population comparisons were handled with -P flag. For example, since we had one male individual from Sarawak and two from Kinabatangan, we ran MSMC2 using -P 0,1,1 when analyzing gene flow between these two populations.

Recent studies in great apes have found signatures of widespread selection affecting the X chromosome [16, 17], which might lead to biased results when estimating effective population sizes based on X-chromosomal data alone. We therefore ran additional MSMC2 analyses using complete autosomal genomes. To avoid coverage related issues, we only used the two individuals from each sampling location with the highest mean genome-wide coverage. We processed the input data in the same manner as the X-chromosomal data and applied identical settings for the MSMC2 runs.

Results were scaled using a X-chromosomal mutation rate of $\mu_X = 1.17 \times 10^{-8}$ mutations per base pair per generation. This was determined using the relationship $\mu_X = (4\mu_A - \mu_Y)/3$, where μ_Y is the Y-chromosomal mutation rate and μ_A is the autosomal mutation rate. This relationship assumes that the autosomal rate is the average of the male and female rates, that the Y-chromosomal rate is equal to the male mutation rate, and that X-chromosomal lineages spend two thirds of their time in females [9]. We assumed an autosomal mutation rate of $\mu_A = 1.5 \times 10^{-8}$ per base pair per generation [1], a Y-chromosomal mutation rate of 2.5×10^{-8} per base pair per generation [18], and used a generation time of 25 years [10].

To further corroborate the results of our gene flow analyses, we fitted a demographic model to the SNP data set using the program momi2 [19]. We devised a model that incorporated population structure on both Borneo and Sumatra and allowed for discrete phases of admixture between the two islands after the initial split (Fig. S8). We used genotype data from three individuals with the highest mean coverage for each of the six populations (two on Sumatra and four on Borneo). To reduce the impact of spurious genotype calls, we applied strict filters for the inclusion of sites into the analysis, requiring three valid genotypes from each population (i.e. no missing data) with a minimum individual coverage of five reads. This resulted in 1,372,932,767 valid sites with 14,037,332 SNPs. We converted the genotypes to a multidimensional site-frequency spectrum using the 'read_vcf.py' and 'extract_sfs.py' scripts of the momi2 package. Using the empirical site-frequency spectrum, we optimized the likelihood of the demographic model in 100 independent runs using the L-BFGS-B algorithm with a maximum number of 1,000 iterations. Based on the highest obtained likelihood, we performed 200 bootstrap resamples from 100 equal-sized data blocks to obtain confidence intervals, using the maximum likelihood estimate of the parameters as starting points of the optimization procedure.

4.2 Results

Cross-population N_e between all Bornean populations and the Sumatran population North Aceh gradually increased to infinity between 1.5 Ma and 400 ka (Fig. S4), suggesting a steady decline of genetic exchange between the two islands during this time frame. However, because MSMC2 is unable to detect sudden changes in gene flow with high precision, actual divergence might have occurred rapidly at some point between these bounds. There appears to be no gene flow between Bornean and North Sumatran populations at more recent times (Fig. S4). Cessation of gene flow between Northeast Alas and all Bornean populations occurred at similar times. No cross-population comparisons with the West Alas population were performed since no male individual from that region was available.

There were signals of divergence between most of the Bornean populations between 40 ka and 20 ka (Figs. 3B and S5). Due to the relative paucity of coalescent events during this period, it is difficult to resolve the order in which the populations diverged from each other. Complex divergence processes would also tend to obscure times of initial separation. Remarkably, we found evidence for gene flow between the Sarawak and Central/West Kalimantan populations and between the North and South Kinabatangan sampling areas continuing to the most recent detectable times (<10 ka, Figs. S5 and S6).

Validation of the cross-population N_e estimates using complete autosomal genomes revealed highly congruent patterns to the analysis limited to X chromosomes only (Fig. S7). However, we note that our estimates of the cross-population N_e on Borneo during the pronounced bottleneck 50-100 ka was smaller by a factor of two to three for the X-chromosome as compared to the autosomes (see Fig. 3B

vs. Fig. S7B). While a small reduction in N_e is expected for the X-chromosomal estimates given the smaller number of X-chromosomes compared to autosomes (0.75 to 1 for equal sex ratios), such a strong reduction might be indicative of widespread effects of selection acting on the X chromosome.

The results from the model fitting using SNP data with momi2 were largely in agreement with our findings from MSMC2 (Table S4). We inferred a split time between Borneo and Sumatra of ~730 ka (95%-CI: 716–743 ka). In agreement with the PSMC and MSMC2 results, momi2 inferred a relatively large ancestral population on Borneo (~28,000 individuals) that went through a bottleneck of ~8,300 individuals between 157 and 25 ka, followed by rapid population divergence. On Sumatra, population sizes increased from ~37,000 to 51,000, before declining to extremely low values in recent times (~95 individuals for each population, Table S4). Contrary to earlier results [1, 20], which inferred population split times on Sumatra on the order of hundreds of thousands of years, momi2 inferred a recent divergence of population at ~1.3 ka, followed by admixture affecting large proportions of the genome. This discrepancy is most likely a result of the limits of the gene flow model implemented in momi2, which only supports discrete admixture events, but not continuous gene flow. Therefore, in the presence of high rates of continuous gene flow, momi2 tends to fit a recent split time between populations on Sumatra. Interestingly, while all MSMC2 runs indicated an infinite cross-population N_e and therefore lack of gene flow between Borneo and Sumatra more recently than ~400 ka, momi2 found evidence for admixture as recent as ~44 ka (Table S4). Even though the estimates of admixture proportions were relatively small (~4.2% from Borneo to Sumatra, ~1.5% from Sumatra to Borneo), both rates were significantly larger than zero and the fit of the model including admixture was significantly better than the corresponding model without admixture (ΔAIC: 100,984.92).

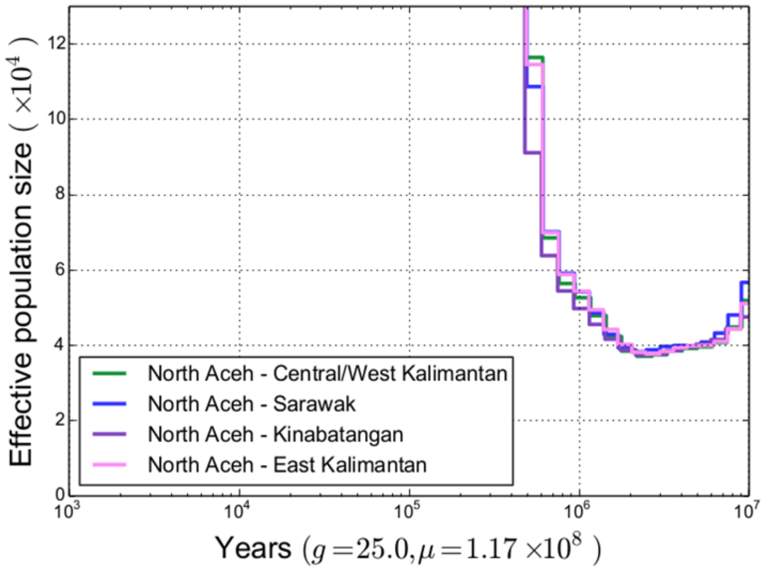

Fig. S4. Gene flow history between North Aceh and Bornean populations. Pseudo-diploid cross-population N_e was inferred using the multiple sequentially Markovian coalescent 2 (MSMC2) on pairs of male X chromosomes from respective populations. The x-axis shows time scaled in years, assuming a generation time of 25 years and an X-chromosomal mutation rate of 1.17×10^{-8} per site per generation. The y-axis shows historical N_e, which is an inverse measure of gene flow between respective populations.

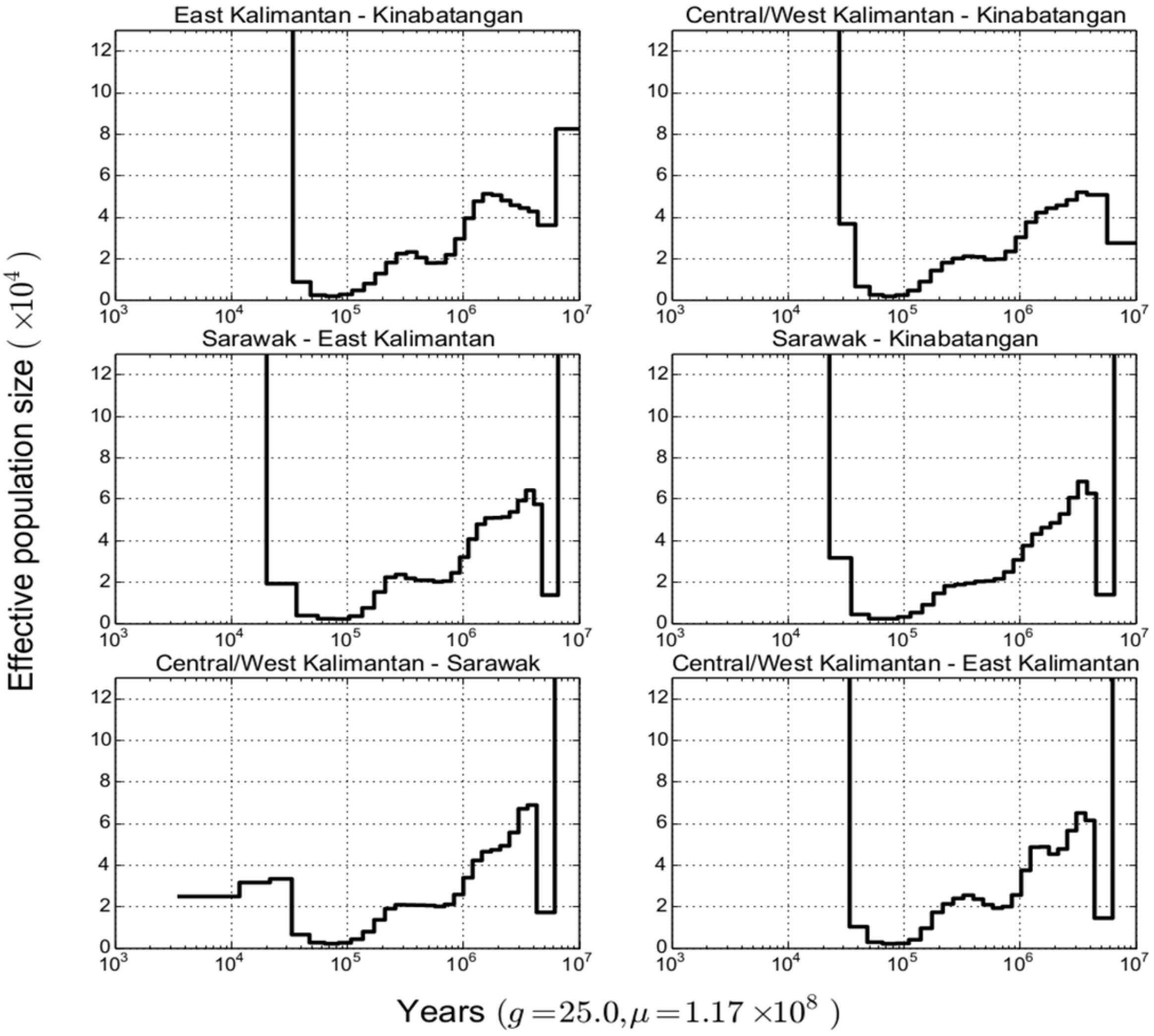

Fig. S5. Gene flow history between Bornean populations. Pseudo-diploid cross-population N_e was inferred using the multiple sequentially Markovian coalescent 2 (MSMC2) on pairs of male X chromosomes from respective populations. The x-axis shows time scaled in years, assuming a generation time of 25 years and an X-chromosomal mutation rate of 1.17×10^{-8} per site per generation. The y-axis shows historical N_e, which is an inverse measure of gene flow between respective populations.

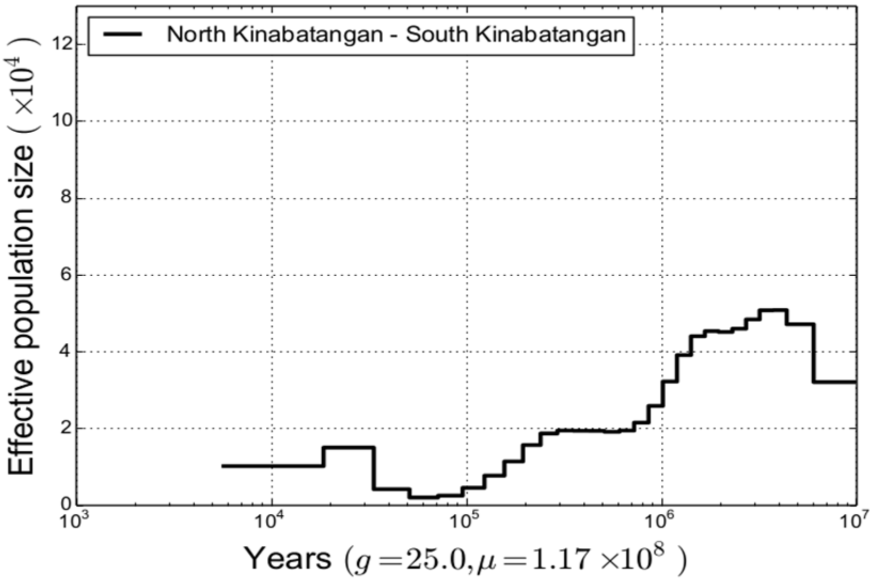

Fig. S6. Gene flow history between North Kinabatangan and South Kinabatangan sampling areas on northeastern Borneo. Pseudo-diploid cross-population N_e was inferred using the multiple sequentially Markovian coalescent 2 (MSMC2) on pairs of male X chromosomes from respective populations. The x-axis shows time scaled in years, assuming a generation time of 25 years and an X-chromosomal mutation rate of 1.17×10^{-8} per site per generation. The y-axis shows historical N_e, which is an inverse measure of gene flow between respective populations.

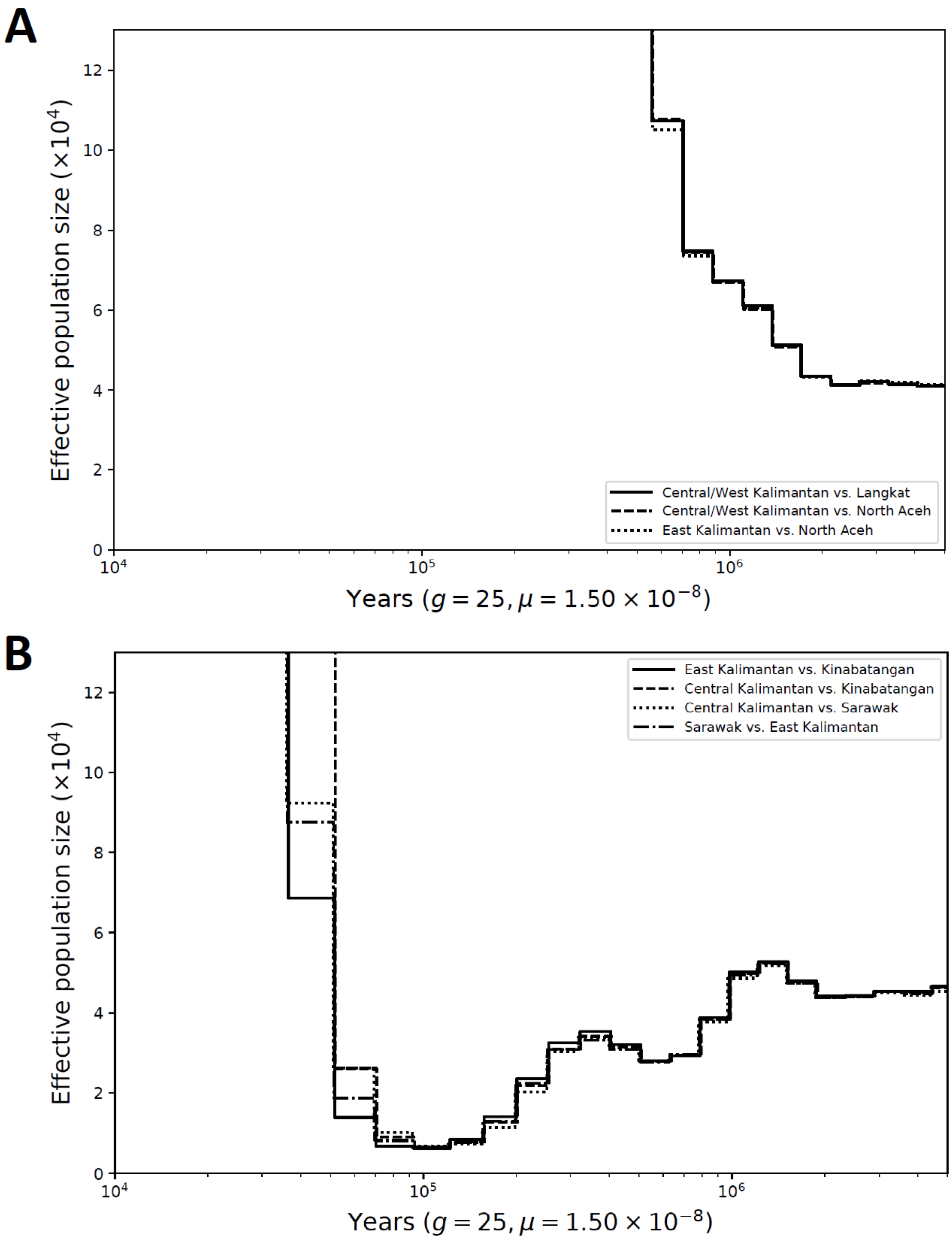

Fig. S7. Validation of gene flow results with phased autosomal data. (A) Temporal estimates of cross-population N_e between population pairs from multiple sequentially Markovian coalescent (MSMC) analyses by comparing autosomal haplotypes of Sumatran populations of Langkat and North Aceh to the Bornean populations of Central/West Kalimantan and East Kalimantan. Cross-population N_e is inversely proportional to gene flow between population pairs, but also influenced by within-population N_e. The x-axis shows time scaled in years, assuming a generation time of 25 years and an autosomal mutation rate of 1.5×10^{-8} per site per generation. (B) Cross-population N_e between Bornean populations estimated from autosomal haplotypes in MSMC, revealing complete genetic isolation from 30 ka onwards for all population pairs.

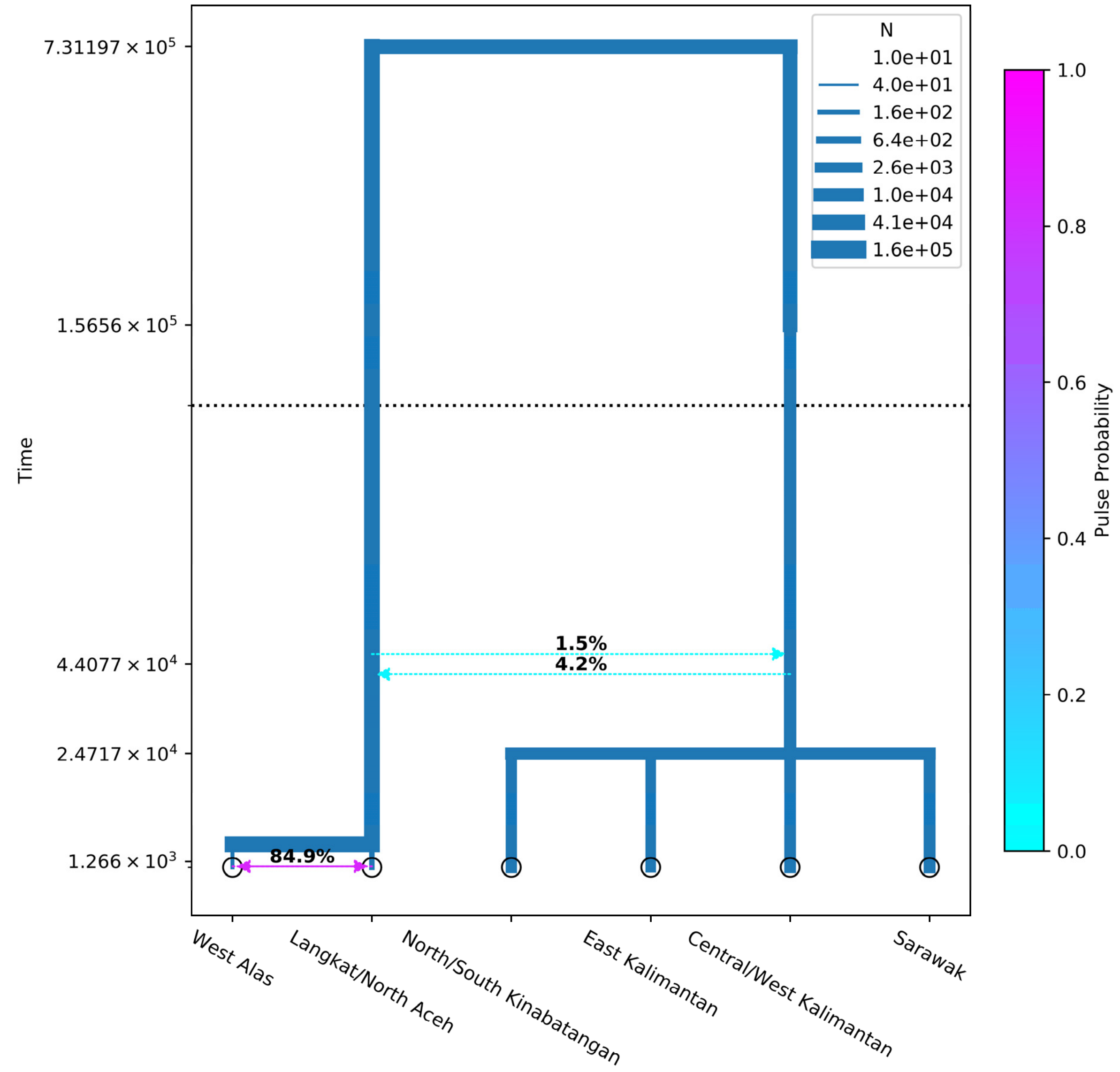

Fig. S8. Illustration of demographic model used for the momi2 analysis. Point estimates of time parameters are shown on the Y-axis, while estimates of effective population size parameters are indicated by the width of population bars. Horizontal arrows depict admixture events and estimated admixture proportions. The Y-axis is linear below the dashed line and follows a logarithmic scale above 100 ka.

Table S4. **Maximum likelihood parameter estimates and bootstrap resampling in the momi2 analysis.**

Parameter[a]	Search bounds[b]	MLE[c]	95%-CI[d]
N_NKSK	1–1,000,000	4,994	4,583–5,492
N_EK	1–1,000,000	3,246	2,960–3,577
N_CKWK	1–1,000,000	5,562	4,987–6,241
N_SR	1–1,000,000	6,454	5,935–7,091
N_LKNA	1–1,000,000	207	188–234
N_WA	1–1,000,000	95	78–113
N_bn_BO	1–1,000,000	8,388	7,197–9,314
N_anc_BO	1–1,000,000	27,935	26,414–29,873
N_anc_SU	1–1,000,000	50,812	48,593–53,156
N_anc_all	1–1,000,000	36,840	36,488–37,306
T_split_BO	1,000–1,000,000	24,717	22,988–26,481
T_bn_BO	1,000–1,000,000	156,560	122,034–190,948
T_split_SU	1,000–1,000,000	1,266	1,239–1,303
T_admix_wSU	100–100,000	194	162–230
T_split_BOSU	10,000–3,000,000	731,197	715,701–743,161
T_admix_BOSU	1,000–1,000,000	44,077	39,469–47,966
P_wSU	0.000–1.000	0.849	0.785–0.898
P_BO_SU	0.000–0.500	0.042	0.040–0.044
P_SU_BO	0.000–0.500	0.015	0.013–0.017

[a], N_XX = effective population size parameters, T_XX = time parameters in years, P_XX_YY = admixture proportions from XX to YY; [b], parameter search ranges for maximum likelihood optimization; [c], maximum likelihood estimate from 100 independent runs; [d], 95% confidence intervals from 200 bootstrap replicates.

Section 5: Mutational load analysis

Bornean orangutans experienced a drastically lower N_e during the Middle Pleistocene (Fig. 2), possibly due to recurrent population bottlenecks during glacial cycles (Figs. 2 and 3B) [1]. Such bottlenecks might have led to an increased fixation of moderately deleterious mutations due to enhanced drift [21]. Evidence for this emerges when correlating the density of segregating deleterious variation in the individual genomes with estimated long-term N_e.

The ratio of non-synonymous to synonymous variants in an individual genome is related to the efficiency of natural selection to remove detrimental variants [22]. We estimated the ratio of stop gain and non-synonymous single nucleotide variants to synonymous variants in each individual, and plotted them against the long-term effective population size (N_e) estimated from Watterson's estimator of *theta* [23]. As expected, and previously described for ten great ape populations [2], those ratios are inversely correlated to effective population size: the smaller the population size the higher proportion of putatively detrimental variants. This correlation is weak and only marginally significant when considering all polymorphic variants within each species (Fig. S9). However, the correlation is strong considering only the variants segregating in each population (Fig. S10), and especially strong for the stop gain variants (Fig. S11). Stop gain mutations are more likely to have a gene-disrupting effect among the loss-of-function variants, while others, such as splice site donor and acceptor variants or start and stop losses, are more difficult to predict. The effect of N_e on the efficiency of natural selection is also seen when representing the number of fixed variants inside each group (species and populations) (Figs. S12 and S13), where species/populations with higher N_e have fewer fixed detrimental variants.

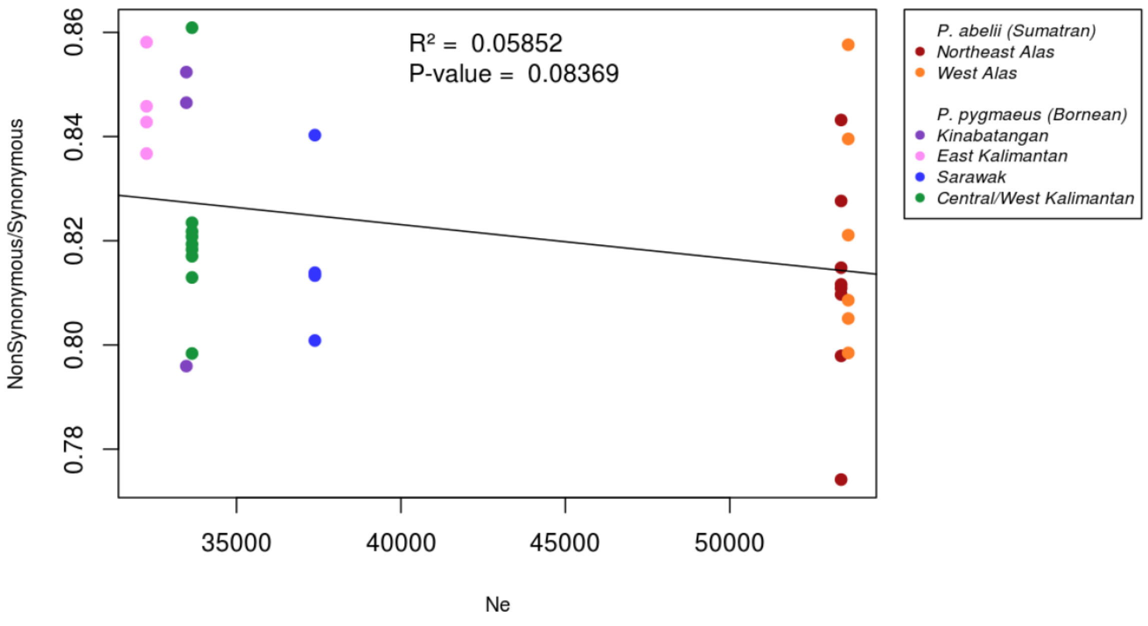

Fig. S9. Ratio of polymorphic non-synonymous to synonymous variants for each individual (y-axis) against long-term effective population size (N_e, x-axis). Color codes match those of Fig. 1. N_e was estimated from the density of segregating sites (Watterson's estimator of theta).

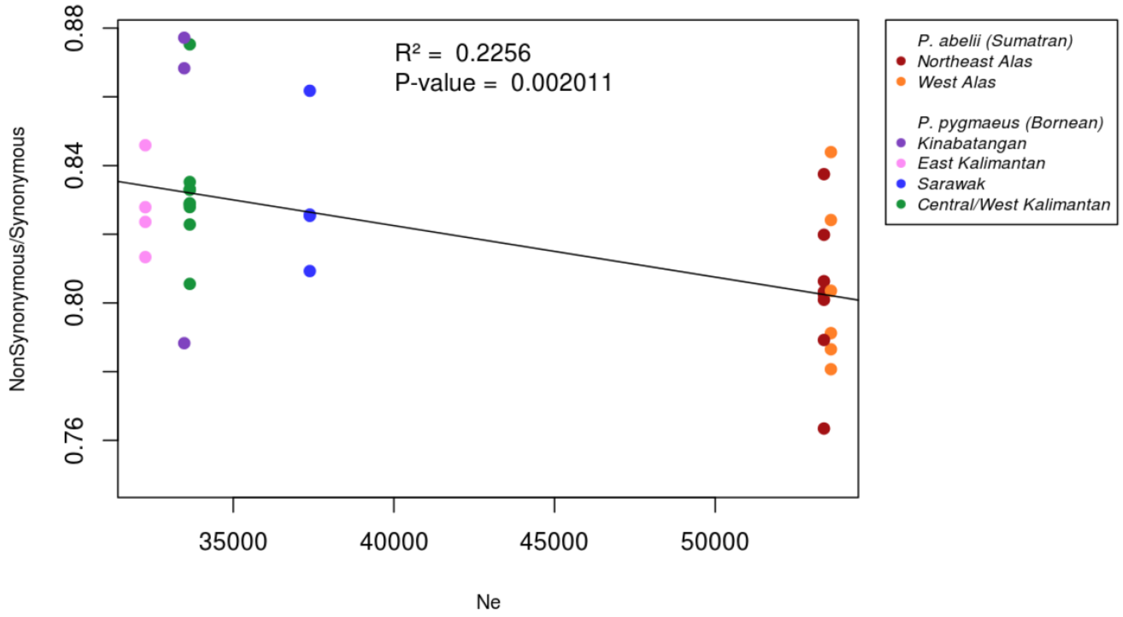

Fig. S10. Ratio of polymorphic non-synonymous to synonymous variants for each individual (y-axis) against long-term effective population size (N_e, x-axis), excluding those variants fixed in each population. Color codes match those of Fig. 1. N_e was estimated from the density of segregating sites (Watterson's estimator of theta).

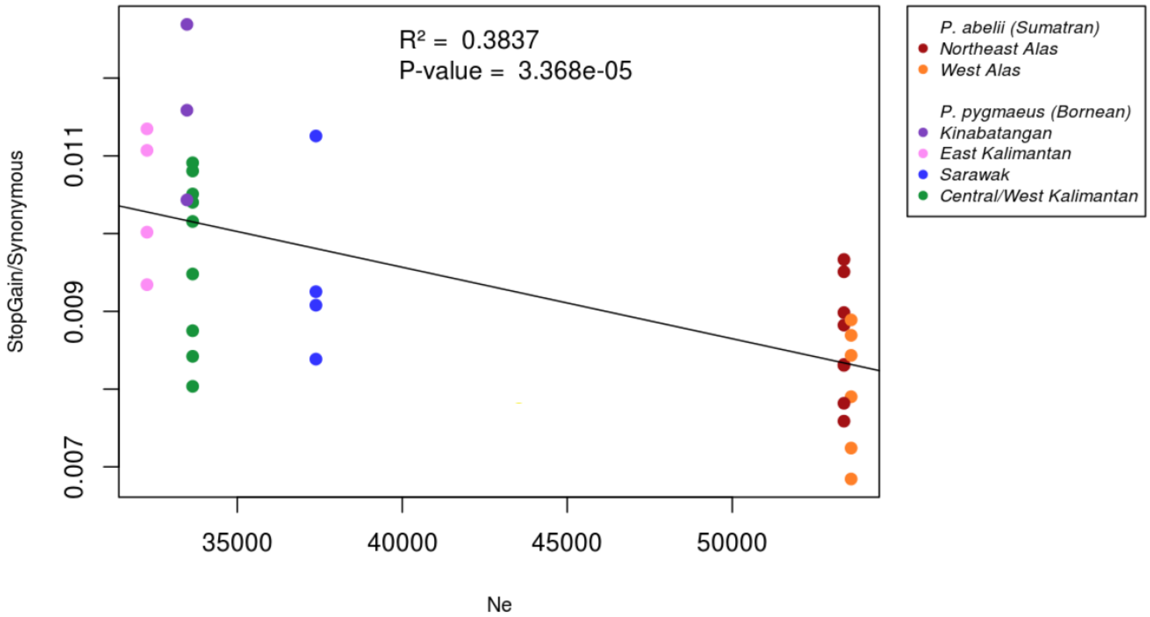

Fig. S11. Ratio of polymorphic stop gain introducing variants to synonymous variants for each individual (y-axis) against long-term effective population size (N_e, x-axis), excluding those variants fixed in each population. Color codes match those of Fig. 1. N_e was estimated from the density of segregating sites (Watterson's estimator of theta).

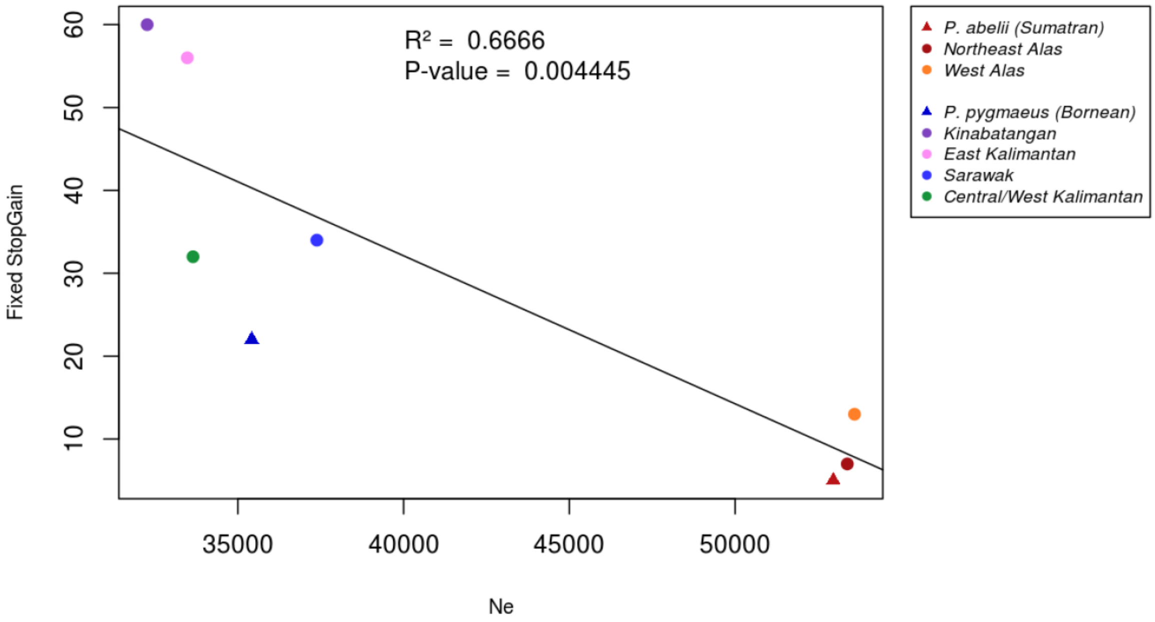

Fig. S12. Absolute number of fixed stop gain introducing variants in each species and population (y-axis) against long-term effective population size (N_e, x-axis). N_e was estimated from the density of segregating sites (Watterson's estimator of theta).

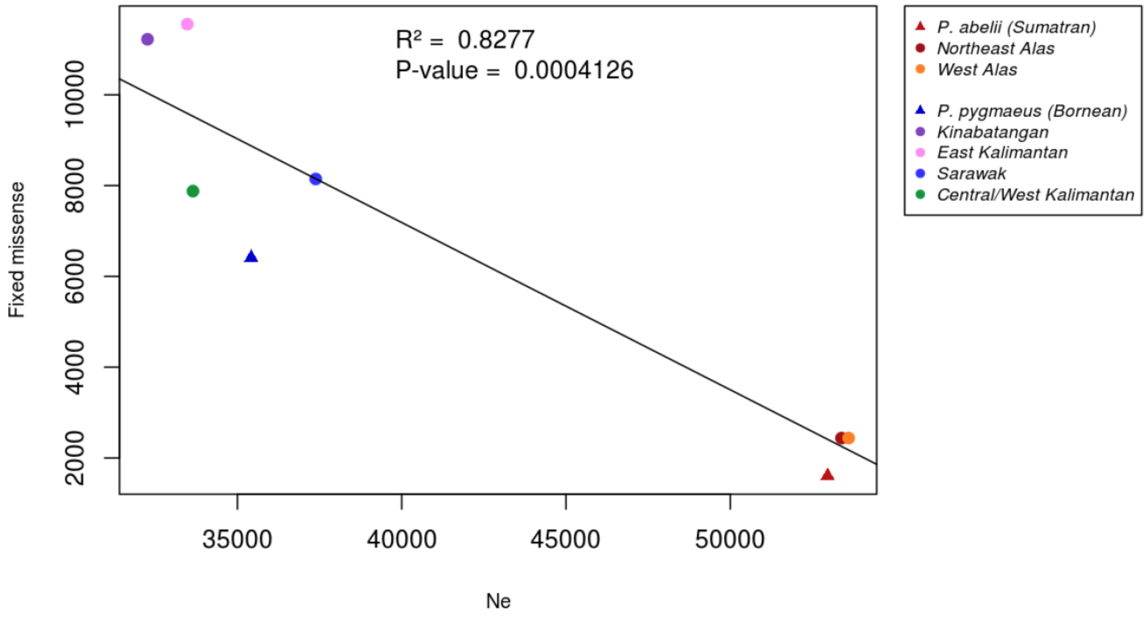

Fig. S13. Absolute number of fixed missense variants in each species and population (y-axis) against long-term effective population size (N_e, x-axis). N_e was estimated from the density of segregating sites (Watterson's estimator of theta).

Section 6: Fixed SNPs between species

6.1 Methods

We investigated fixed SNPs between species in more detail, i.e. SNPs for which all Bornean orangutans were homozygous for one allele and all North Sumatran orangutans for the other. We only considered SNP positions which were covered by at least 10 genotypes per species with a minimal sequence depth of 5x (n = 27,037,765 SNPs) and identified fixed SNPs with custom R scripts. To characterize the effects of fixed SNP variants on genes, transcripts, and protein sequence we used the Variant Effect Predictor [24] implemented in the Ensembl genome browser (http://www.ensembl.org/info/docs/tools/vep/). For all genes containing at least one non-synonymous fixed SNP, we again obtained detailed functional information with GeneALaCart.

We performed GO enrichment analyses for fixed SNPs between species. For these SNP-based GO analyses, we used the program GOWINDA [25]—a software that was designed for genome-wide association studies. Classical GO analyses may be biased as longer genes typically have more SNPs, thus a higher probability of being sampled [25]. Permutation tests implemented in GOWINDA take this into account and allow for an unbiased analysis of gene set enrichment [25]. To obtain an orangutan-specific gene set file of GO terms, we downloaded biological process GO terms for all protein-coding genes in the orangutan genome from the BioMart web-interface (accessed April 5th 2015) and converted the file to the format required by GOWINDA in R. The list of candidate genes was built from genes which contained at least one fixed SNP within a window of 5,000 bp upstream and downstream of the gene. Including these flanking regions ensured to capture also SNPs within close-by regulatory elements [26, 27]. The background list of genes for significance assessment was derived from all variable sites used to identify the fixed SNPs. We applied the recommended more conservative '–gene flag', which assumes that all SNPs within a gene are completely linked. Significance thresholds ($P < 0.05$) after false-discovery rate (FDR) correction were obtained empirically based on 100,000 simulations.

6.2 Results

Out of 27,037,765 analyzed autosomal SNPs, 123,023 SNPs (0.455%) were completely fixed for different alleles in Bornean and North Sumatran orangutans (Additional file 2: Table S13). These SNPs constitute the genetic basis of differences between the two orangutan species, which might have arisen either by random genetic drift or directional selection. Of all fixed SNPs, 39.9% were located within 5 kb of a protein-coding gene, indicating an enrichment of fixed SNPs in gene and regulatory regions (Additional file 2: Table S14). Gene ontology analysis of protein-coding genes containing fixed SNPs (3,889 genes) revealed statistically significant (P FDR < 0.05) enrichment of 19 biological GO categories (Table S5, Additional file 2: Table S15). The significantly enriched GO terms were associated with brain development (n = 2), skeletal development (n = 3), metabolism (n = 5), organismal development (n = 4), and regulation of transcription (n = 3). Enriched gene ontologies include for example two terms possibly associated with differences in diet between Bornean and North Sumatran orangutans, i.e. the sensory perception of taste and the response to stilbenoid. Stilbenoids are a class of plant phenolics occurring in the wood and fruits of several plant families, including tropical Dipterocarpaceae and Gnetaceae [28]. Orangutan fallback food is higher in phenolics than fruit pulp and seeds [29].

Among the 123,023 SNPs fixed between Bornean and North Sumatran orangutans, 296 SNPs were non-synonymous, i.e. altered amino acids, and three were splice donor/acceptor variants (Additional file 2: Table S14). A proportion of these SNPs likely represent causal variants underlying phenotypic differences between the two orangutan species. Fixed non-synonymous SNPs altered 236 protein-coding genes, of which 28 were uncharacterized novel genes in *Pongo* (identified by Ensembl Gene Build). Two fixed non-synonymous SNPs resulted in gain of a premature stop codon (loss-of-function mutations) in the genes *ARGFX* and *ZNF224*. *ARGFX* is a putative transcription factor and thought to be involved in early embryonic development. *ZNF224* may be involved in transcriptional regulation as repressor. We identified further loss-of-function mutations in splicing regions, which affected the gene *SRBD1* (splice donor variant), whose function remains unknown, and two uncharacterized novel genes (ENSPPYG00000010361, ENSPPYG00000000950). We did not find significant (P FDR < 0.05) enrichment of genes with potential functional changes for any particular biological GO term. Detailed functional and disease association information of all genes containing fixed non-synonymous SNPs or splice acceptor/donor variants are provided in Additional file 2: Table S16.

Table S5. **Significantly enriched gene ontology (GO) terms between North Sumatran and Bornean orangutans.** Listed GO terms were significantly enriched in the analysis of SNPs fixed between species. We report only GO terms that are related to biological processes.

GO term	GO description	*P* FDR[a]	No. of genes[b]
**Brain development**			
GO:0021797	Forebrain anterior/posterior pattern specification	0.00165	5/5
GO:0021938	Smoothened signaling pathway involved in regulation of cerebellar granule cell precursor cell proliferation	0.04976	4/4
**Skeletal development**			
GO:0048706	Embryonic skeletal system development	0.01067	19/39
GO:0060348	Bone development	0.01067	26/38
**Metabolism**			
GO:0050909	Sensory perception of taste	0.00605	14/31
GO:0051453	Regulation of intracellular pH	0.04083	6/7
GO:0072001	Renal system development	0.04632	11/18
GO:2000377	Regulation of reactive oxygen species metabolic process	0.02304	12/17
GO:0007275	Multicellular organismal development	0.00165	88/188
GO:0009952	Anterior/posterior pattern specification	0.00165	41/87
GO:0009953	Dorsal/ventral pattern formation	0.03875	23/43
GO:0061154	Endothelial tube morphogenesis	0.04868	4/4
**Regulation**			
GO:0000122	Negative regulation of transcription from RNA Polymerase II promoter	0.01637	181/460
GO:0045944	Positive regulation of transcription from RNA Polymerase II promoter	0.00165	280/702
GO:0030178	Negative regulation of Wnt signaling pathway	0.01067	24/42

[a]*P*-value after adjustment for multiple testing; [b]the number of unique genes found for the given GO term related to the total number of genes that could be found at most for this term, i.e. genes that have a corresponding entry in the annotation file and contain at least one SNP.

Section 7: Codon models for positive selection analysis

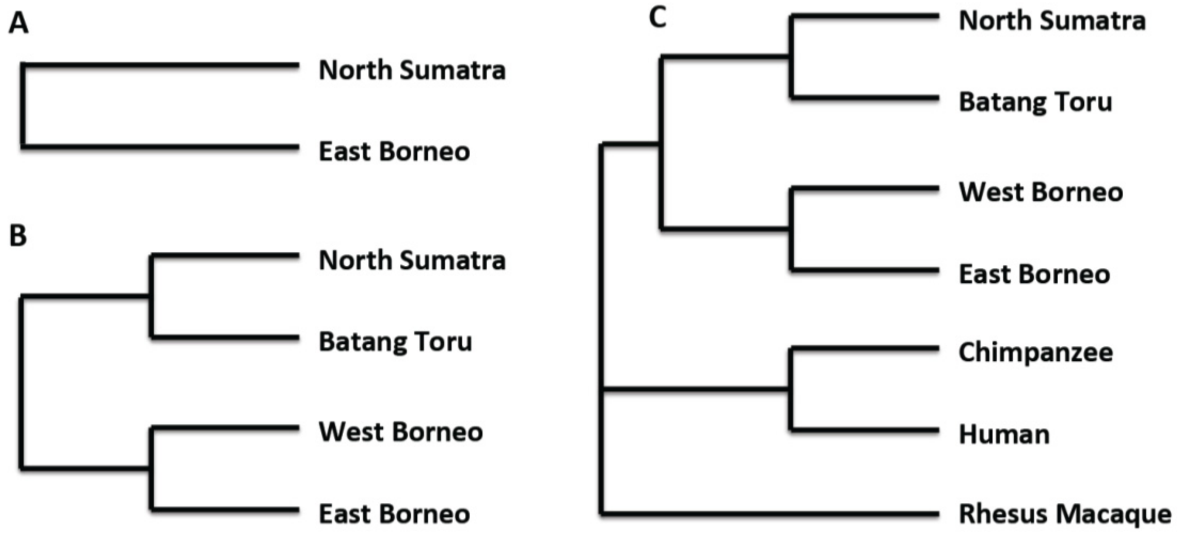

Fig. S14. Species topologies used in the codon modeling. The North Sumatran clade contains all *P. abelii* individuals except those from Batang Toru. The East Bornean clade includes all *P. pygmaeus morio* individuals, whereas the remaining Bornean individuals belong to the West Bornean clade. The outgroup species include the human, chimpanzee and rhesus macaque lineages. Because some genes might exhibit gene trees incongruent with the assumed species topology, we filtered out the exons that have low branch support (<0.5 aBayes support) [30] at the internal branch separating Sumatran from Bornean individuals in the unrooted tree setting.

Table S6. Genes with evidence of positive selection in Northeast Borneo. Genes are grouped based on the pathways or functions they are associated to according to GeneCards database.

HUGO Gene ID	Function based on interaction network analysis	2Δl LRT
**Muscle activity**		
CDON	myogenesis	2.799
EFCAB9	muscle contraction	3.285
GAS2L2	crosslink microfilaments in muscle	3.113
MLYCD	fatty acid biosynthesis in muscle	3.076
NAIP	modifier of spinal muscular atrophy	3.755
NRAP	muscle tendon junction	3.822
RGS20	cardiac muscle activity	4.100
TNNI3	cardiac muscle relaxation, obesity, diabetes	3.354
TNNI3K	cardiac muscle contraction	3.354
**Heart and vessels**		
EFNA5	activates the EPHA3	2.751
EPHA3	cardiac cell differentiation	5.780
GLG1	interactions at the vascular wall	3.797
JAG1	heart development	3.108
**Fertilization**		
CMPK1	nucleotide metabolism, ovulation	3.768
EFCAB6	spermatogenesis and fertilization	3.285
LIMK2	spermatogenesis	3.851
MUC16	cell surface signaling in ovaries	4.700
SPATA13	spermatogenesis	5.420
TEKT3	sperm mobility	3.243
BRCA1	cell cycle, estragen pathway	3.226
PES1	main regulator of estrogen levels	4.678
**Olfaction**		
OR5AU1	olfactory perception	3.232
OR6D2	olfactory perception	3.197
OR7D2	olfactory perception	3.454
**Peroxisome metabolism**		
FAT1	peroxisome proliferation, regulate PEX5 and TG activity	13.409
PEX5	peroxisomal biogenesis	3.388
TG	insulin secretion	3.008
**Lipid metabolism**		
FPGT	glycolipid metabolism	6.776
GPR116	fat cell differentiation	5.469
LGSN	glutamine synthesis	6.471
**Hyaluronan metabolism**		
ABCC5	hyaluronic acid biosynthesis	2.829
ACAN	binds to hyaluronic acid	4.218
STAB2	binds to hyaluronic acid, glycogen storage	3.630
**Brain and nervous system**		
KIF1C	reelin pathway, brain development	9.949
RELN	cortex development	2.819
SPTA1	actin cytoskeleton, neural growth	3.294
**Hair**		
KRT23	hair follicle	4.267
KRT32	hair keratin	6.431
**Other**		
CAPN14	apoptosis, cell division	2.800
FRAS1	organogenesis during development	3.863
GRK7	visual perception	3.083
IGF2R	regulates fetal growth	2.790
PARP15	transcription regulation	3.191
PDZD2	pain pathway	4.042
ZNF236	transcription regulation	2.912
ZNF777	transcription regulation	3.054
MCM10	DNA replication	6.181

Table S7. Genes with evidence of positive selection in North Sumatra.

HUGO Gene ID	Function based on interaction network analysis	2Δl LRT
**Brain and nervous system**		
ADGRF3	neuropeptide signaling	3.197
DLC1	neural tube closure	3.062
HAUS2	mitotic activity in brain	3.123
MPDZ	postsynaptic activity	3.584
MTCH1	neuronal ion channel	3.180
NEK1	axonal development	3.208
NTRK2	neuron migration and survival	5.336
PLCB1	signaling in brain and neural tube	5.157
RECQL5	energy metabolism in cerebral cortex	3.399
SF3A3	embryonic development of the brain	4.060
SH3RF2	embryonic development of the neural tube	3.907
SMC2	mitotic activity in brain	4.152
SYT1	synaptic neurotransmitter release in brain	3.701
TENM2	neural development	3.233
YARS	neuronal signal transduction in brain	3.961
**Kidney**		
RPGRIP1L	kidney development	2.910
SLC12A1	kidney development	5.770
**Metabolism**		
ABCA13	phospholipid transport	3.040
ABCC1	lipid metabolism	3.874
ABCC2	glucose/energy metabolism	2.758
ACO2	glucose/energy metabolism	2.925
LPIN1	fatty acid, triacylglycerol and ketone body metabolism	2.880
LYPLAL1	lipid metabolism	3.940
PIGR	protein-energy metabolism	3.656
SAMM50	protein metabolism in mitochondria	3.812
SLC9B2	energy metabolism	4.189
**Other**		
GAS8	cell growth	4.359
PDCD11	cell death	3.617
SERPINA4	immune system	3.391
SERPING1	immune system	3.421
TAS2R38	taste receptor	2.745
ZFP161	transcription regulation	5.238

We repeated the MCM positive selection tests for the genes with the evidence of positive selection by using the whole protein coding transcripts (Table S8). We merged the exons that fulfil the original criteria for each gene using the bedtools.

Table S8. Results of MCM using whole protein coding transcripts.

Northeast Borneo		North Sumatra	
**HUGO Gene ID**	**2Δl**	**HUGO Gene ID**	**2Δl**
ABCC5	4.66016	ABCA13	3.04027
ACAN	4.21728	ABCC1	3.87419
BRCA1	3.22596	ABCC2	2.75766
CAPN14	2.79917	ACO2	4.99823
CDON	4.49756	ADGRF3	3.19737
CMPK1	7.91556	CACNB2	4.39668
CNTN2	5.66427	DLC1	3.06256
DMXL2	3.23381	GAS8	3.24736
EFCAB6	3.28472	HAUS2	3.12283
EFNA5	2.75127	LPIN1	3.95157
EPHA3	5.77908	LYPLAL1	3.94015
FAM184A	9.4647	MPDZ	3.58354
FAT1	13.4094	MTCH1	3.18018
FPGT	6.77569	NEK1	4.13116
FRAS1	3.86342	NTRK2	5.33643
GAS2L2	3.11336	PDCD11	3.61745
GLG1	3.79665	PIGR	3.65565
GPR116	22.296	PLCB1	5.15674
GRK7	3.93602	RECQL5	3.39903
IGF2R	2.78969	RPGRIP1L	2.90991
JAG1	18.90462	SAMM50	8.45200
KIF1C	9.49883	SERPINA4	3.39107
KRT23	17.06778	SERPING1	3.42126
KRT32	6.43136	SF3A3	4.05953
LGSN	6.47103	SH3RF2	3.90674
LIMK2	3.85154	SLC12A1	5.77033
MAP3K10	3.3365	SLC9B2	4.18922
MCM10	6.18065	SMC2	9.94144
MLYCD	5.54278	SYT1	3.70096
MUC16	4.70021	TAS2R38	5.51144
NAIP	3.75485	TENM2	3.23282
NAV2	4.84717	YARS	3.96115
NRAP	3.82186	ZFP161	5.23814
OR5AU1	11.75782		
OR6D2	3.19736		
OR7D2	22.779		
PARP15	3.1909		
PDZD2	4.04203		
PES1	4.67827		
PEX5	6.54676		
RASL11A	3.03538		
RELN	2.81864		
RGS20	4.09976		
SPATA13	16.18588		
SPTA1	2.7276		
STAB2	3.62969		
TEKT3	3.24328		
TG	3.00826		
TNNI3K	3.3543		
TNNI3K	3.3543		
ZNF236	2.91207		
ZNF777	3.0535		

Section 8: Interaction network analyses

To gain a better understanding of the functions of genes putatively under positive selection in the two orangutan species, we employed a network approach to visualize interaction relationships among the sets of genes identified in Section 7.

We generated gene networks using GeneMania App 3.4.0 in Cytoscape 3.3.0 [31]. We used the functional association database of GeneMania to retrieve the interaction networks within the set of candidate genes. We disabled the search for relevant genes, so that each node in the network was a candidate gene identified through MCM. Genes were labeled with the HUGO gene ID.

For candidate genes found in East Borneo (n = 46) and in North Sumatra (n = 33) in the codon modeling approach, we considered all interaction classes except the predicted interactions class. We found that most of the candidate genes were involved in one of the following interaction classes: co-expression, genetic interaction, shared pathway, shared protein domain, physical interaction. The genes that were not involved in any interaction with other genes in the sets are shown as single nodes in the networks.

We assigned functional classes to genes according to the presence of specific keywords in the GeneCards encyclopedia summaries of gene function and disease association annotations from the following databases: Entrez Gene of the National Center for Biotechnology Information (NCBI), UniProt Knowledgebase (UniProtKB/Swiss-Prot), The Human Malady Compendium (MalaCards), and DISEASES database (Table S9). Different functional classes were coded by different colors.

Table S9. Keywords used for functional classification of candidate genes.

Functional class	Keywords
Lipid metabolism	lipid, lipo-, fat, adipo-, peroxi-, obesity, diabet-, insulin
Glucose metabolism	sugar, carbo-, gluco-
Brain and neural development	nerv-, neurogenesis, neurite, axon guidance, cortex, cereb-
Learning and memory	learning, memory, synaptic plasticity, long-term potentiation, post synaptic synapse formation
Cardiac/muscle activity	cardi-, muscle, myo-, atrioventricular
Stress response	environmental stress, stress
Cellular growth	cell cycle, mito-, survival, cellular growth, proliferation, replicati-, death, division
Reproduction	reproduction, fertilization, germinal, gamete, sperm, ovary, -male
Immune response	immune response, inflammatory response

The classification of the keyword search was manually checked for each gene. In case of multiple putative functions and/or a lack of a main function, we did not assign the gene to a functional class. Following these procedures, we could assign at least half of the candidate genes in each set to one of the functional classes listed in Table S9.

Section 9: Population-based selection analyses

We also carried out population-based selection analyses to uncover candidate loci that might explain the genetic basis of phenotypic differences observed among populations within the two orangutan species. We applied two complementary tests in genome-wide windows to uncover signatures of ongoing or recent selective sweeps. First, we ran a composite likelihood ratio (CLR) test to assess site frequency spectra to investigate local frequency shifts relative to the genomic background. Second, we applied a haplotype-based selection test (iHS) [32] occurring within the last few thousand years [33]. In both approaches, we identified significant departure from patterns expected under neutrality by running neutral coalescent simulations under the demographic model described in Nater et al. [1].

9.1 Methodological considerations

There are two methodological challenges that might bias our population-based selection analyses. First, low sequencing coverage of certain individuals sequenced here might bias the discovery rate of SNPs against low-frequency variants, which will affect our selection tests. However, we believe that our methods are robust to such a bias. The iHS statistic was developed and tested for the analysis of chip-array data [32], which usually show some degree of ascertainment bias, similar to what is expected to occur with non-uniform coverage in whole-genome sequencing. Furthermore, iHS is based on the relative length of haplotypes carrying the ancestral and derived alleles, thus it is considered to be quite robust to biases affecting the allele frequency spectrum [32].

The second methodological challenge is related to the relatively low sample sizes of the studied populations. However, sample sizes are to be considered when simulating the neutral *null* model, resulting in a broader *null* distribution of test statistics and therefore reduced power to detect outliers, but no apparent bias.

9.2 Data sets for population-level analyses

We used genome-wide SNP data from 30 individuals from four populations to perform population-level tests of positive selection (Table S10). For each individual, we estimated the haplotypes at each chromosome using SHAPEIT as described in Section 2. Additionally, we determined the ancestral state for each SNP (Section 2). We excluded sites that were not biallelic, for which the ancestral state could not be determined, or which were monomorphic at the population level.

Table S10. Samples used for population-level selection analyses.

Species	Population	Individuals
P. abelii	Northeast Alas	PA_KB4661, PA_KB5883, PA_A947, PA_A948, PA_A950, PA_A952, PA_A949, PA_B018
	West Alas	PA_KB4361, PA_SB550, PA_B017, PA_A953, PA_A955, PA_B020
P. pygmaeus	Central/West Kalimantan (*P. p. wurmbii*)	PP_KB4204, PP_KB5404, PP_KB5405, PP_A940, PP_A941, PP_A943, PP_A944, PP_A938, PP_A983
	Kinabatangan/ East Kalimantan (*P. p. morio*)	PP_KB5543, PP_A984, PP_A985, PP_A987, PP_A988, PP_5062, PP_A989

9.3 Tests for positive selection

We calculated the composite likelihood ratio (CLR) test statistic using the program SweeD v3.3.2 [34]. We applied a spacing of grid points for the calculation of the CLR statistic of 12.5 kb, using the unfolded site-frequency spectrum. To ensure accurate allele frequency estimates across genomic regions with varying coverage, we required genotypes to be covered by at least five reads, and sites to have at least 80% of individuals with valid genotypes.

We used the R package *rEHH* [35] with default parameters to obtain absolute iHS scores for each informative polymorphic site in each population, using only polymorphic sites with no missing genotypes in all individuals of a given population. We then averaged iHS scores in sliding windows of 25 kb with 12.5 kb step size (iHS_{avg}). Averaging absolute iHS scores in genomic windows reduces the variance of the statistic and it is commonly advised [32, 36].

9.4 Neutral simulations

In order to assess thresholds of significance for each population, we performed a set of neutral simulations based on the demographic model estimated through ABC modeling described in Nater et al. [1] by sampling model parameter values from the posterior density distributions. For each population, we matched the sample sizes of the simulations to the empirical data. We fixed the mutation rate at 1.5×10^{-8} per base pair per generation [1], and sampled the recombination rate for each simulated locus from a uniform distribution between 10^{-9} and 10^{-8} per base pair per generation (covering approximately the range observed during recombination map estimation, see Nater et al. [1]).

In total, we generated 10,000 replicates, simulating sequences spanning 100 kb, and analyzed the simulated data with the same pipeline as described above. We used the same sliding window parameters as for the empirical data to average iHS scores and the same grid point spacing as for the calculation of the CLR statistic, allowing us to check for possible simulation biases along the simulated sequence. Since we did not observe any bias towards the ends of the simulated sequences, we used all the test scores from the simulated 100 kb sequences to build the score distributions and establish 1%-FPR thresholds. By applying the thresholds estimated according to the neutral *null* model, we identified a set of regions under putative positive selection for each population.

9.5 Candidate gene information and GO enrichment analysis

We identified protein-coding genes located within putative sweep regions with the BioMart web-interface [37] of the Ensembl genome browser (http://www.ensembl.org/biomart/), searching the '*Pongo abelii* genes' dataset (Ensembl release 84). We further gathered detailed information on identified protein-coding genes using GeneALaCart (LifeMap Sciences, Inc.), which allows extracting information on a large number of genes from the GeneCards encyclopedia—an integrated database of information dealing with human genes (https://genealacart.genecards.org; last accessed March 19th 2016) [38]. We obtained GeneCards summaries of gene function and disease association annotations from the following major knowledge databases: Entrez Gene of the National Center for Biotechnology Information (NCBI), UniProt Knowledgebase (UniProtKB/Swiss-Prot), The Human Malady Compendium (MalaCards), and DISEASES database (disease-gene associations mined from literature).

To examine whether genes within putative selective sweep regions (i.e. candidate genes) were enriched for any particular biological process, we performed an analysis of Gene Ontology (GO) terms using the R package 'gProfileR' of the g:Profiler toolkit [39, 40]. Significance was assessed by comparing the candidate genes with a background list of all possible genes, i.e. all protein-coding genes (n = 12,866) located within any window with sufficient coverage for calculation of the iHS and CLR statistics. We applied the Benjamini-Hochberg method [41] for computing multiple testing correction for *P*-values gained from GO enrichment analysis.

9.6 Overlap of candidate genes among populations

Our gene mapping strategy provided an approximate genome-wide measure of the number of genes in regions showing evidence of positive selection, as well as an estimate of the overlap among populations. Overlap of identified candidate genes between populations was generally low, indicating geographically localized recent sweep events (Table S11 and S12). As expected, comparisons of populations within each species showed a generally higher overlap as compared to population pairs between species.

Table S11. Overlap among populations of candidate genes identified in the iHS tests.

	Northeast Alas	West Alas	Kinabatangan/East Kalimantan	Central/West Kalimantan
**Northeast Alas**	350	-	-	-
**West Alas**	28	319	-	-
**Kinabatangan/East Kalimantan**	8	9	216	-
**Central/West Kalimantan**	11	10	22	256

Table S12. Overlap among populations of candidate genes identified in the CLR tests.

	Northeast Alas	West Alas	Kinabatangan/East Kalimantan	Central/West Kalimantan
**Northeast Alas**	456	-	-	-
**West Alas**	108	409	-	-
**Kinabatangan/East Kalimantan**	37	35	295	-
**Central/West Kalimantan**	54	51	84	346

References

1. Nater A, Mattle-Greminger MP, Nurcahyo A, Nowak M, de Manuel M, Desai T, Groves C, Pybus M, Bilgin Sonay T, Roos C *et al*. Morphometric, behavioral, and genomic evidence for a new orangutan species. Current Biology. 2017;27(22):3487-3498.
2. Prado-Martinez J, Sudmant PH, Kidd JM, Li H, Kelley JL, Lorente-Galdos B, Veeramah KR, Woerner AE, O/'Connor TD, Santpere G *et al*. Great ape genetic diversity and population history. Nature. 2013;499(7459):471-475.
3. Locke DP, Hillier LW, Warren WC, Worley KC, Nazareth LV, Muzny DM, Yang S-P, Wang Z, Chinwalla AT, Minx P *et al*. Comparative and demographic analysis of orang-utan genomes. Nature. 2011;469(7331):529-533.
4. Li H, Durbin R. Fast and accurate short read alignment with Burrows–Wheeler transform. Bioinformatics. 2009;25(14):1754-1760.
5. McKenna A, Hanna M, Banks E, Sivachenko A, Cibulskis K, Kernytsky A, Garimella K, Altshuler D, Gabriel S, Daly M *et al*. The Genome Analysis Toolkit: A MapReduce framework for analyzing next-generation DNA sequencing data. Genome Research. 2010;20(9):1297-1303.
6. DePristo MA, Banks E, Poplin R, Garimella KV, Maguire JR, Hartl C, Philippakis AA, del Angel G, Rivas MA, Hanna M *et al*. A framework for variation discovery and genotyping using next-generation DNA sequencing data. Nat Genet. 2011;43(5):491-498.
7. Greminger MP, Stolting K, Nater A, Goossens B, Arora N, Bruggmann R, Patrignani A, Nussberger B, Sharma R, Kraus RH *et al*. Generation of SNP datasets for orangutan population genomics using improved reduced-representation sequencing and direct comparisons of SNP calling algorithms. BMC genomics. 2014;15(1):16.
8. Delaneau O, Marchini J, Zagury JF. A linear complexity phasing method for thousands of genomes. Nat Methods. 2012;9(2):179-181.
9. Li H, Durbin R. Inference of human population history from individual whole-genome sequences. Nature. 2011;475(7357):493-496.
10. Wich S, De Vries H, Ancrenaz M, Perkins L, Shumaker R, Suzuki A, Van Schaik C. Orangutan life history variation. In: *Orangutans - Geographic Variation in Behavioral Ecology and Conservation* Edited by Wich SA, Utami Atmoko SS, Mitra Setia T, van Schaik CP: Oxford University Press 2009: 65-75.
11. Scally A, Durbin R. Revising the human mutation rate: implications for understanding human evolution. Nature Reviews Genetics. 2012;13(10):745-753.
12. Ségurel L, Wyman MJ, Przeworski M. Determinants of Mutation Rate Variation in the Human Germline. Annual Review of Genomics and Human Genetics. 2014;15(1):47-70.
13. Venn O, Turner I, Mathieson I, de Groot N, Bontrop R, McVean G. Strong male bias drives germline mutation in chimpanzees. Science. 2014;344(6189):1272-1275.
14. Lipson M, Loh P-R, Sankararaman S, Patterson N, Berger B, Reich D. Calibrating the human mutation rate via ancestral recombination density in diploid genomes. PLoS Genet. 2015;11(11):e1005550.
15. Chesner C, Rose W, Deino A, Drake R, Westgate J. Eruptive history of Earth's largest Quaternary caldera (Toba, Indonesia) clarified. Geology. 1991;19(3):200-203.
16. Nam K, Munch K, Hobolth A, Dutheil JY, Veeramah KR, Woerner AE, Hammer MF, Mailund T, Schierup MH. Extreme selective sweeps independently targeted the X chromosomes of the great apes. Proceedings of the National Academy of Sciences. 2015.
17. Dutheil JY, Munch K, Nam K, Mailund T, Schierup MH. Strong selective sweeps on the X chromosome in the human-chimpanzee ancestor explain its low divergence. PLoS genetics. 2015;11(8):e1005451.
18. Xue Y, Wang Q, Long Q, Ng BL, Swerdlow H, Burton J, Skuce C, Taylor R, Abdellah Z, Zhao Y *et al*. Human Y chromosome base-substitution mutation rate measured by direct sequencing in a deep-rooting pedigree. Curr Biol. 2009;19(17):1453-1457.

19. Kamm JA, Terhorst J, Durbin R, Song YS. Efficiently inferring the demographic history of many populations with allele count data. bioRxiv. 2018.
20. Nater A, Greminger MP, Arora N, van Schaik CP, Goossens B, Singleton I, Verschoor EJ, Warren KS, Krützen M. Reconstructing the Demographic History of Orang-utans using Approximate Bayesian Computation. Molecular Ecology. 2015;24(2):310-327.
21. Henn BM, Botigue LR, Peischl S, Dupanloup I, Lipatov M, Maples BK, Martin AR, Musharoff S, Cann H, Snyder MP *et al*. Distance from sub-Saharan Africa predicts mutational load in diverse human genomes. Proceedings of the National Academy of Sciences of the United States of America. 2016;113(4):E440-E449.
22. Lohmueller KE, Indap AR, Schmidt S, Boyko AR, Hernandez RD, Hubisz MJ, Sninsky JJ, White TJ, Sunyaev SR, Nielsen R *et al*. Proportionally more deleterious genetic variation in European than in African populations. Nature. 2008;451(7181):994-U995.
23. Watterson G. On the number of segregating sites in genetical models without recombination. Theoretical population biology. 1975;7(2):256-276.
24. McLaren W, Pritchard B, Rios D, Chen Y, Flicek P, Cunningham F. Deriving the consequences of genomic variants with the Ensembl API and SNP Effect Predictor. Bioinformatics. 2010;26(16):2069-2070.
25. Kofler R, Schlötterer C. Gowinda: unbiased analysis of gene set enrichment for genome-wide association studies. Bioinformatics. 2012;28(15):2084-2085.
26. Blanchette M, Bataille AR, Chen X, Poitras C, Laganière J, Lefèbvre C, Deblois G, Giguère V, Ferretti V, Bergeron D *et al*. Genome-wide computational prediction of transcriptional regulatory modules reveals new insights into human gene expression. Genome Research. 2006;16(5):656-668.
27. ENCODE Project Consortium. An integrated encyclopedia of DNA elements in the human genome. Nature. 2012;489(7414):57-74.
28. Sotheeswaran S, Pasupathy V. Distribution of resveratrol oligomers in plants. Phytochemistry. 1993;32(5):1083-1092.
29. Leighton M. Modeling dietary selectivity by Bornean orangutans: evidence for integration of multiple criteria in fruit selection. International Journal of Primatology. 1993;14(2):257-313.
30. Anisimova M, Gil M, Dufayard JF, Dessimoz C, Gascuel O. Survey of branch support methods demonstrates accuracy, power, and robustness of fast likelihood-based approximation schemes. Syst Biol. 2011;60(5):685-699.
31. Shannon P, Markiel A, Ozier O, Baliga NS, Wang JT, Ramage D, Amin N, Schwikowski B, Ideker T. Cytoscape: a software environment for integrated models of biomolecular interaction networks. Genome Res. 2003;13(11):2498-2504.
32. Voight BF, Kudaravalli S, Wen X, Pritchard JK. A map of recent positive selection in the human genome. PLoS Biol. 2006;4(3):e72.
33. Sabeti PC, Schaffner SF, Fry B, Lohmueller J, Varilly P, Shamovsky O, Palma A, Mikkelsen TS, Altshuler D, Lander ES. Positive natural selection in the human lineage. Science. 2006;312(5780):1614-1620.
34. Pavlidis P, Živković D, Stamatakis A, Alachiotis N. SweeD: Likelihood-Based Detection of Selective Sweeps in Thousands of Genomes. Molecular Biology and Evolution. 2013;30(9):2224-2234.
35. Gautier M, Vitalis R. rehh: an R package to detect footprints of selection in genome-wide SNP data from haplotype structure. Bioinformatics. 2012;28(8):1176-1177.
36. Pybus M, Luisi P, Dall'Olio GM, Uzkudun M, Laayouni H, Bertranpetit J, Engelken J. Hierarchical boosting: a machine-learning framework to detect and classify hard selective sweeps in human populations. Bioinformatics. 2015;31(24):3946-3952.
37. Kasprzyk A. BioMart: driving a paradigm change in biological data management. Database. 2011;2011:bar049.
38. Safran M, Dalah I, Alexander J, Rosen N, Stein TI, Shmoish M, Nativ N, Bahir I, Doniger T, Krug H. GeneCards Version 3: the human gene integrator. Database. 2010;2010:baq020.
39. Reimand J, Kull M, Peterson H, Hansen J, Vilo J. g: Profiler—a web-based toolset for functional profiling of gene lists from large-scale experiments. Nucleic acids research. 2007;35(suppl 2):W193-W200.

40. Reimand J, Arak T, Vilo J. g: Profiler—a web server for functional interpretation of gene lists (2011 update). Nucleic acids research. 2011;39(suppl 2):W307-W315.
41. Benjamini Y, Hochberg Y. Controlling the false discovery rate: a practical and powerful approach to multiple testing. Journal of the royal statistical society Series B (Methodological). 1995:289-300.
